# Phosphoric acid-etched boron nitride as a single-atom support for boosting CO_2_ electroreduction at high current density

**DOI:** 10.1039/d6sc06494a

**Published:** 2026-10-12

**Authors:** Xuan Li, Yu-Ting Wang, Wei-Zhen Deng, Qian-Run Tan, Hong-Mei Liu, Wen-Juan Huang, Li-Dong Wang, Zi-Xuan Wu, Yuan-Jing Suo, Yan-Tong Xu, Li-Ming Cao, Hai-Hua Huang, Chun-Ting He

**Affiliations:** a Key Lab of Fluorine and Silicon for Energy Materials and Chemistry of Ministry of Education, College of Chemistry and Materials, Jiangxi Normal University Nanchang 330022 China caolm@jxnu.edu.cn huanghh@jxnu.edu.cn hct@jxnu.edu.cn; b School of Advanced Energy, Sun Yat-Sen University (Shenzhen) Shenzhen 518107 China

## Abstract

Designing adjustable and robust support materials remains a critical challenge for enhancing the industrial-level CO_2_ electroreduction performance of single-atom catalysts. Herein, we report a defective and phosphate-modified boron nitride (BN) support *via* a facile phosphoric acid etching strategy to anchor single nickel atoms. The optimized electrocatalyst delivers an outstanding maximum CO faradaic efficiency of 97.6 ± 1.2% in an H-cell and a large CO partial current density of 148.6 ± 2.3 mA cm^−2^ in a gas diffusion electrode-based flow cell and retains high CO selectivity during 87 h of stable operation. A variety of characterization studies including *operando* spectroscopy combined with theoretical calculations reveal that phosphoric acid etching redistributes the electronic structures of the interface as well as Ni centers, which significantly optimizes the *CO adsorption. Moreover, the surface phosphate species may play a critical role in stabilizing the key *COOH intermediate. This work not only provides a support material for achieving performance gain of transition metal single atoms, but also offers new insights into interfacial molecule-mediated CO_2_ electrochemical conversion.

## Abstract 2

A defective and phosphate-modified boron nitride support boosts efficient electrocatalytic CO_2_ reduction with Ni single atoms.

## Graphical abstract

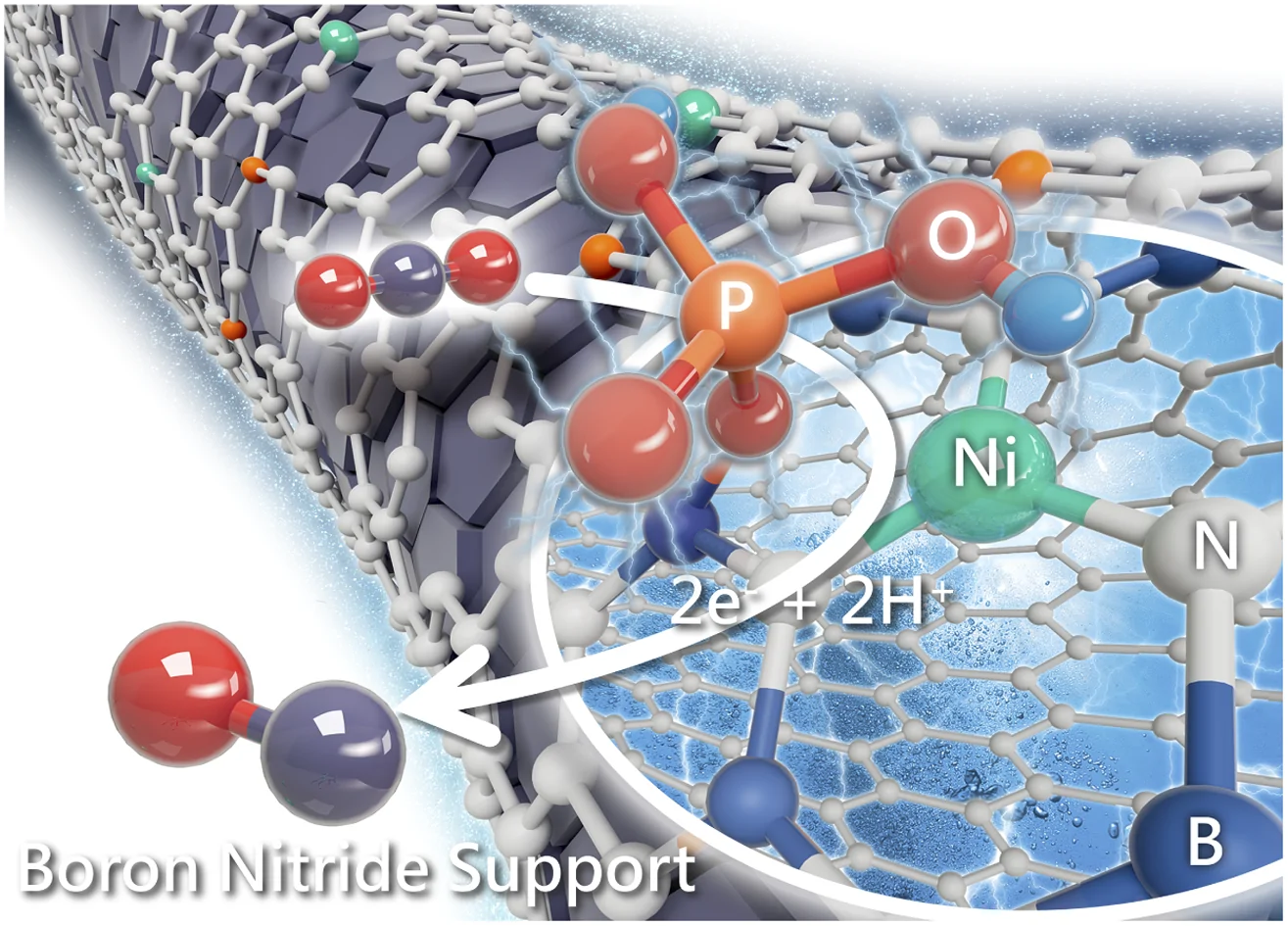

## Introduction

The escalating global climate change and energy shortages have driven extensive research into viable technologies for converting greenhouse gas carbon dioxide (CO_2_) into high-value chemicals and fuels.^1–3^ Among these, the electrochemical CO_2_ reduction reaction (CO_2_RR) stands out as a sustainable route, which employs renewable electricity to transform CO_2_ into carbon monoxide (CO), formic acid, methane and other products, holding tremendous practical application prospects.^4–7^ In particular, the selective conversion of CO_2_ to CO has attracted widespread research interest, as CO acts as an indispensable C1 platform molecule for industrial manufacturing while simultaneously mitigating excessive CO_2_ emissions.^8–11^

Nevertheless, the practical implementation of the CO_2_RR is hindered by multiple bottlenecks, such as the high energy barrier for CO_2_ activation,^12^ severe competing hydrogen evolution reaction (HER),^13–15^ and unsatisfactory selectivity toward target carbonaceous products.^16,17^ To address these obstacles, it is critical to develop electrocatalysts with high activity, outstanding selectivity and robust long-term durability.^18–20^ Single-atom catalysts (SACs) have become appealing contenders for the CO_2_RR, thanks to their special geometric arrangement, maximized atomic efficiency and excellent catalytic properties.^21–25^ Loading isolated metal atomic species onto proper support materials offers an effective route to tailor the electronic properties and local coordination environment of active centers, further altering the reaction routes and product selectivity toward the CO_2_RR.^26–30^ Nevertheless, the practical performances of SACs are often compromised by the inadequate anchoring stability and support robustness, especially at high current densities. Under such working conditions, SACs are highly prone to agglomeration, while traditional carbon-based or oxide supports are also susceptible to corrosion or structural degradation, severely limiting the activity and stability of the catalytic system.

To address these issues, boron nitride (BN) materials, particularly boron nitride nanotubes (BNNTs) and boron nitride networks (BNNs), are regarded as promising candidates for SAC supports owing to their excellent chemical inertness, large specific surface area and tunable electronic properties.^31,32^ BN exhibits outstanding corrosion resistance and high structural stability at high potentials in electrocatalysis. However, the electrical conductivity of boron nitride is relatively poorer compared to that of carbon materials. Fortunately, recently, studies have shown that hybridizing BN networks with carbon materials *via* an appropriate strategy would significantly improve the conductivity of the materials while maintaining their structural integrity and robustness.^33,34^ Moreover, structural defect engineering enables enhanced metal loading and the construction of strong bonding sites.^35^ Nevertheless, the rational manipulation of the defects and electronic characteristics of the BN substrate for boosting catalytic performances of anchored single atoms remains a critical challenge.^36–38^

In this work, we report a phosphoric-acid etching strategy to remould the surface microenvironment of BN. The etching produces defects and modifies phosphate species on the BN coated carbon nanotubes (CNTs), which serve as supports to anchor Ni single atoms, resulting in a catalyst of Ni_0.05_/P-eBN@CNT.^39,40^ The phosphate species can strengthen the interfacial interaction between Ni single atoms and the support and optimize the electronic configuration of Ni centers.^41^ In an H-cell, Ni_0.05_/P-eBN@CNT delivers exceptional CO selectivity with a maximum faradaic efficiency (FE_CO_) of 97.6 ± 1.2%. In a gas diffusion electrode (GDE) flow cell, the catalyst delivers a high FE_CO_ of 91.5 ± 1.7% at a high current density of 150 mA cm^−2^. Experimental characterization and density functional theory (DFT) calculations reveal that the defective BN can modulate the electronic structure of Ni sites, while the surface phosphorus-containing moieties may stabilize the critical *COOH intermediate *via* intermolecular hydrogen bonds, thereby lowering the energy barrier of the key step for CO_2_-to-CO conversion.^42,43^ This work offers a tunable catalyst support together with theoretical guidance for designing efficient SACs toward sustainable CO_2_RR.

## Results and discussion

### Structural characterization

The BN@CNT composite was first synthesized by the high-temperature pyrolysis of urea, boric acid and amino-functionalized CNTs, followed by phosphoric acid etching to generate rich surface defects. The obtained P-eBN@CNT substrate could capture different amounts of Ni^2+^ cations *via* defective N sites to form the Ni-containing precursor (Fig. 1). Finally, Ni_0.05_/P-eBN@CNT was prepared by thermal treatment of the precursor. For comparative investigation of the effects induced by phosphoric acid treatment, Ni_0.05_/B-eBN@CNT was prepared using boric acid-etched BN@CNT (B-eBN@CNT) as a support. Transmission electron microscopy (TEM) images reveal that an ultrathin (BN) layer is uniformly coated on the outer walls of CNTs (Fig. 2a and S1). Raman spectra simultaneously detect the D band of CNTs at 1320 cm^−1^ and the characteristic E_2g_ vibrational peak of BN at 1351 cm^−1^ (Fig. S2).^44^ Combined with the characteristic stretching absorption of B–N bonds observed at 1363 cm^−1^*via* Fourier-transform infrared spectroscopy (FTIR) (Fig. S3),^45^ the three characterization studies collectively verify the successful fabrication of integrated BN@CNT conductive supports. The aberration-corrected high-angle annular dark-field scanning transmission electron microscopy (AC-HAADF-STEM) image (Fig. 2b) reveals that Ni_0.05_/P-eBN@CNT exhibits numerous isolated, discrete bright spots, corresponding to atomic Ni sites. Corresponding energy-dispersive X-ray spectroscopy (EDS) elemental mapping (Fig. 2c) demonstrates that C, B, N, P and Ni are uniformly distributed throughout the entire nanotube framework, indicating the presence of phosphorus species. Inductively coupled plasma mass spectrometry (ICP-MS) quantitative measurements determine the P content to be 0.183 wt% and the Ni loading to be 0.297 wt% in Ni_0.05_/P-eBN@CNT (Table S1).

**Fig. 1:**
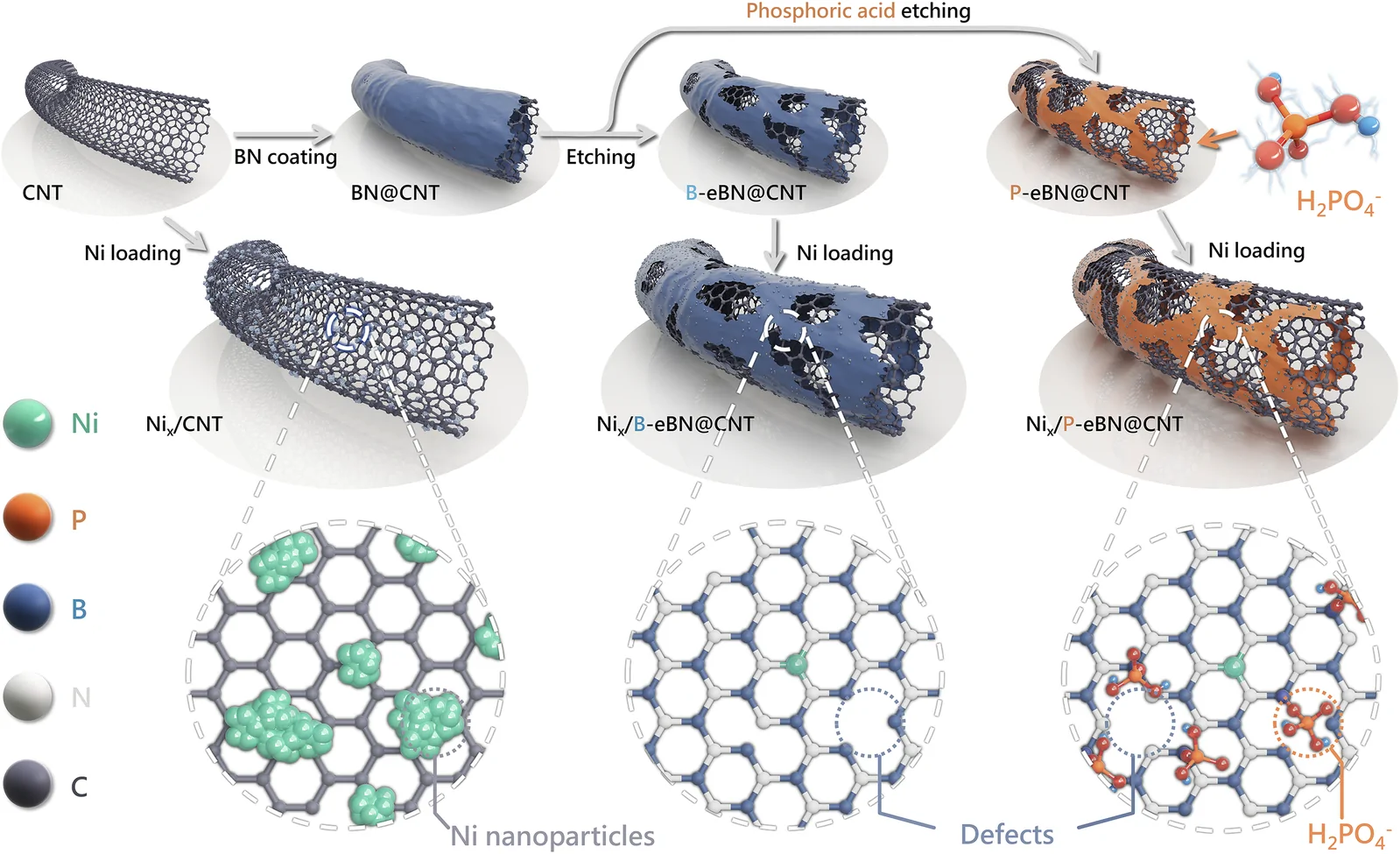
Synthetic schematic of Ni_*x*_/CNT, Ni_*x*_/B-eBN@CNT and Ni_*x*_/P-eBN@CNT catalysts. BN layers were grown on CNTs to form BN@CNT, which was further etched using boric acid or phosphoric acid to produce B-eBN@CNT and P-eBN@CNT. Compared with boric-acid etching, phosphoric-acid etching can simultaneously introduce defects and surface phosphate species. Ni nanoparticles or single atoms were immobilized on the three different substrates to prepare target catalysts.

**Fig. 2:**
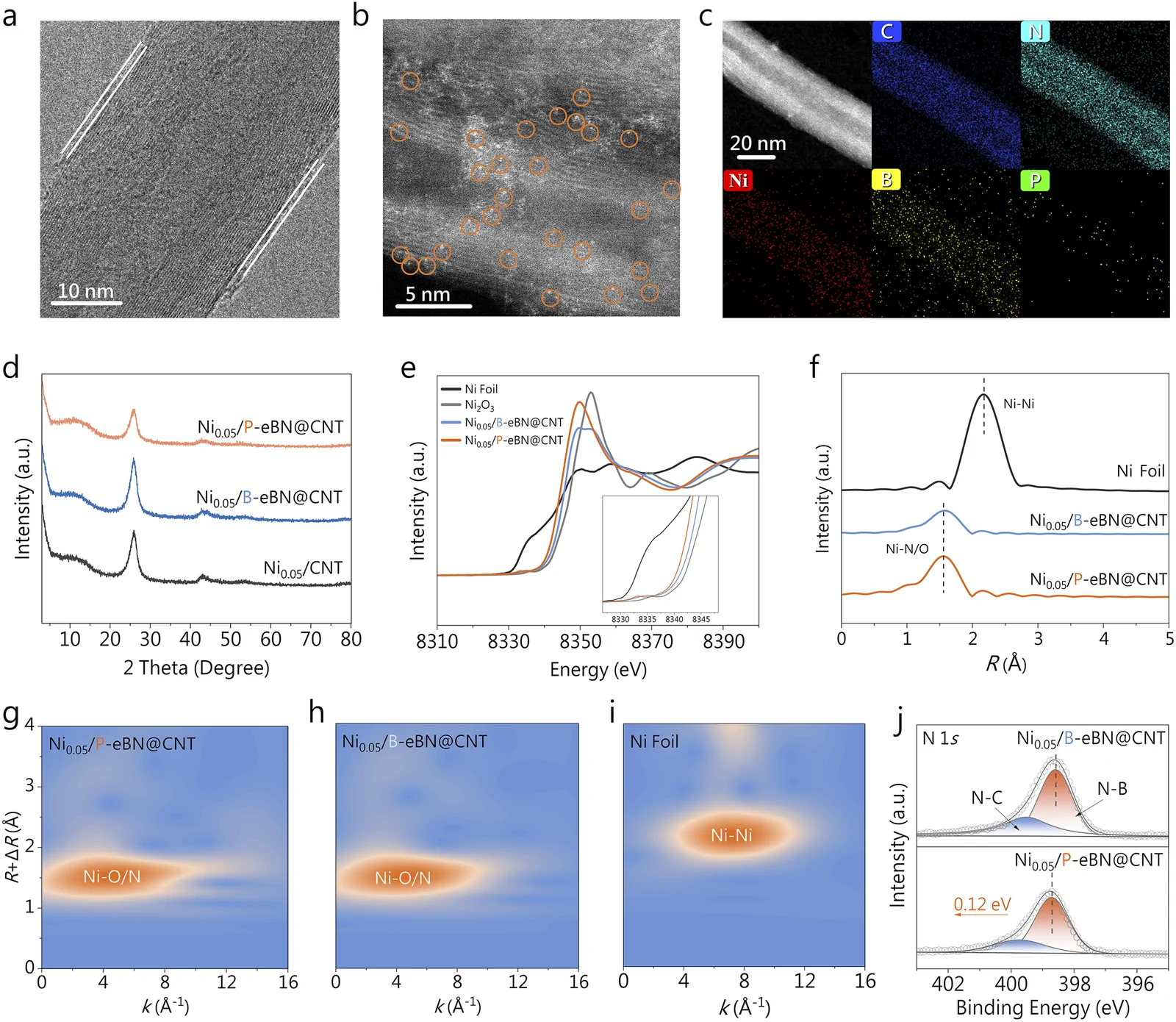
Structural characterization. (a) TEM images of Ni_0.05_/P-eBN@CNT. (b) AC-HAADF-STEM images of Ni_0.05_/P-eBN@CNT. (c) EDX elemental mapping images of Ni_0.05_/P-eBN@CNT. (d) PXRD patterns of Ni_0.05_/P-eBN@CNT, Ni_0.05_/B-eBN@CNT and Ni_0.05_/CNT. (e) Ni K-edge XANES spectra. (f) FT-EXAFS spectra of Ni_0.05_/P-eBN@CNT, Ni_0.05_/B-eBN@CNT, Ni foil and Ni_2_O_3_. Wavelet transform contour plots of *k*^2^-weighted EXAFS spectra of (g) Ni_0.05_/P-eBN@CNT, (h) Ni_0.05_/B-eBN@CNT and (i) Ni foil reference, respectively. (j) High-resolution XPS spectra of N 1s for Ni_0.05_/B-eBN@CNT and Ni_0.05_/P-eBN@CNT.

Powder X-ray diffraction (PXRD) patterns reveal that acid etching weakens the crystallinity of BN networks in both BN@CNT and P-eBN@CNT substrates (Fig. 2d and S4). After Ni immobilization, none of the three catalysts exhibit detectable diffraction peaks assigned to crystalline Ni species. Only the typical (002) and (100) characteristic reflections of BN are observed for Ni_0.05_/P-eBN@CNT, Ni_0.05_/B-eBN@CNT and the BN-free catalyst Ni_0.05_/CNT, demonstrating the high dispersion of Ni species on the supports.^46^ Furthermore, the BN diffraction peaks of Ni_0.05_/P-eBN@CNT present lower peak intensity and slightly broadened full width at half maximum (FWHM), which coincides with the severe distortion of the BN E_2g_ peak in its Raman profile. These results suggest that phosphoric acid etching introduces a higher concentration of B/N vacancies and supplies sufficient unsaturated coordination sites for anchoring metal atoms.

To precisely elucidate the chemical state of Ni anchored on BN networks, X-ray absorption spectroscopy (XAS) measurements were performed to analyze the local coordination configuration and electronic properties of Ni. Ni K-edge X-ray absorption near-edge structure (XANES) spectra (Fig. 2e) show a negative shift of the absorption edge for Ni_0.05_/P-eBN@CNT relative to Ni_0.05_/B-eBN@CNT, which points to a lower average oxidation state of the Ni atomic sites.^47^ Fourier-transform extended X-ray absorption fine structure (FT-EXAFS) spectra (Fig. 2f) reveal that no evident Ni–Ni metallic bonding signals comparable to those of the Ni foil reference can be observed for both BN-supported catalysts at ∼2.48 Å, indicating that Ni exists as single-atom sites.^48^ Quantitative fitting yields a Ni–N coordination number of 2.74 ± 0.22 and Ni–N bond length of 2.04 ± 0.01 Å for Ni_0.05_/P-eBN@CNT. For Ni_0.05_/B-eBN@CNT, the Ni–N coordination number is 2.73 ± 0.15 with a bond length of 2.02 ± 0.01 Å (Table S2). Wavelet-transform EXAFS contour plots (Fig. 2g–i) further rule out the existence of Ni–Ni bonding in both catalysts and only exhibit contour signals assigned to Ni–N coordination. High-resolution X-ray photoelectron spectroscopy (XPS) measurements were performed to analyze the interfacial interaction and electronic configuration of the two catalysts. For Ni_0.05_/P-eBN@CNT, its deconvoluted B 1s spectrum resolves three characteristic peaks located at 191.10 eV, 192.00 eV and 192.72 eV, which are assigned to B–C, B–N and B–O bonds, respectively (Fig. S5).^49^ Meanwhile, the C 1s spectra can be split into three components centered at 284.67 eV (C–B), 284.80 eV (C–C) and 285.66 eV (C–N) (Fig. S6).^50^ The coexistence of B–C and C–N bonds solidly confirms the successful coating of BN layers on CNT backbones, accompanied by strong interfacial bonding between BN and CNTs.^51^ Moreover, deconvolution of N 1s spectra yields two characteristic peaks at ∼398.59 eV for N–B bonds and ∼399.58 eV for N–C bonds, and the whole N 1s envelope of Ni_0.05_/P-eBN@CNT displays a 0.12 eV shift toward higher binding energy relative to Ni_0.05_/B-eBN@CNT (Fig. 2j). This positive binding energy shift indicates that the abundant B/N vacancies and surface phosphorus species introduced by phosphoric acid etching may induce a net redistribution of electron density from the BN framework to the Ni center.^52,53^ Overall, the structure discrepancy between the two etching protocols is systematically elucidated across multiple scales, covering microscopic coating morphology, lattice defect density, atomic coordination geometry and interfacial electron redistribution.

### Electrocatalytic performance evaluation

We first conducted comparative studies on the intrinsic catalytic activity of all catalysts in an H-type electrolytic cell. To screen the optimal metal doping ratio, three samples Ni_0.03_/B-eBN@CNT, Ni_0.05_/B-eBN@CNT and Ni_0.1_/B-eBN@CNT with different Ni loadings were prepared and subjected to constant-potential electrolysis at −0.85 V *versus* RHE. Comparative measurements reveal that Ni_0.05_/B-eBN@CNT delivers the best performance with a CO faradaic efficiency (FE) of 88.4 ± 0.5% (Fig. S7). Therefore, all comparative catalysts were synthesized at a fixed feeding mass ratio of Ni to the support of 0.05.

After determining the optimal Ni loading, three catalysts of Ni_0.05_/P-eBN@CNT, Ni_0.05_/B-eBN@CNT and BN-free Ni_0.05_/CNT with identical metal loadings were fabricated to systematically compare the electrocatalytic performances. Linear sweep voltammetry (LSV) profiles demonstrate that the two defective BN-supported catalysts exhibit far larger cathodic reduction current densities than Ni_0.05_/CNT (Fig. S8), among which the phosphoric acid-etched catalyst shows the best activity. In terms of product selectivity, Ni_0.05_/P-eBN@CNT achieves a maximum CO FE of 97.6 ± 1.2% at −0.9 V *versus* RHE, outperforming Ni_0.05_/B-eBN@CNT (88.4 ± 0.5%). Both defective BN-based catalysts exhibit good CO selectivity, while severe competing HER occurred on BN-free Ni_0.05_/CNT, leading to a drastically decreased CO FE of only 58.9 ± 1.6% (Fig. 3a). Moreover, the partial current density of CO (*j*_CO_) further verified the superior CO production capability of Ni_0.05_/P-eBN@CNT at −1.05 V *versus* RHE, which delivered the maximum *j*_CO_ of 21.3 ± 0.4 mA cm^−2^, exceeding the values of Ni_0.05_/B-eBN@CNT (17.3 ± 0.3 mA cm^−2^) and Ni_0.05_/CNT (5.7 ± 0.2 mA cm^−2^) (Fig. S9). Electrochemical double-layer capacitance (*C*_dl_) and electrochemical impedance spectroscopy (EIS) further validated the above performance disparities. The *C*_dl_ value of Ni_0.05_/P-eBN@CNT is 13.87 mF cm^−2^, which is higher than those of Ni_0.05_/B-eBN@CNT (6.23 mF cm^−2^), Ni_0.05_/CNT (6.11 mF cm^−2^) and the bare P-eBN@CNT support (2.30 mF cm^−2^) (Fig. S10 and S11). Meanwhile, Ni_0.05_/P-eBN@CNT displays the smallest charge-transfer semicircle in the EIS plots (Fig. S12). The above results indicate that the defects generated by phosphoric acid etching contribute to an increased density of accessible active sites and accelerated charge transfer, thereby promoting the electrocatalytic CO_2_RR performance.

**Fig. 3:**
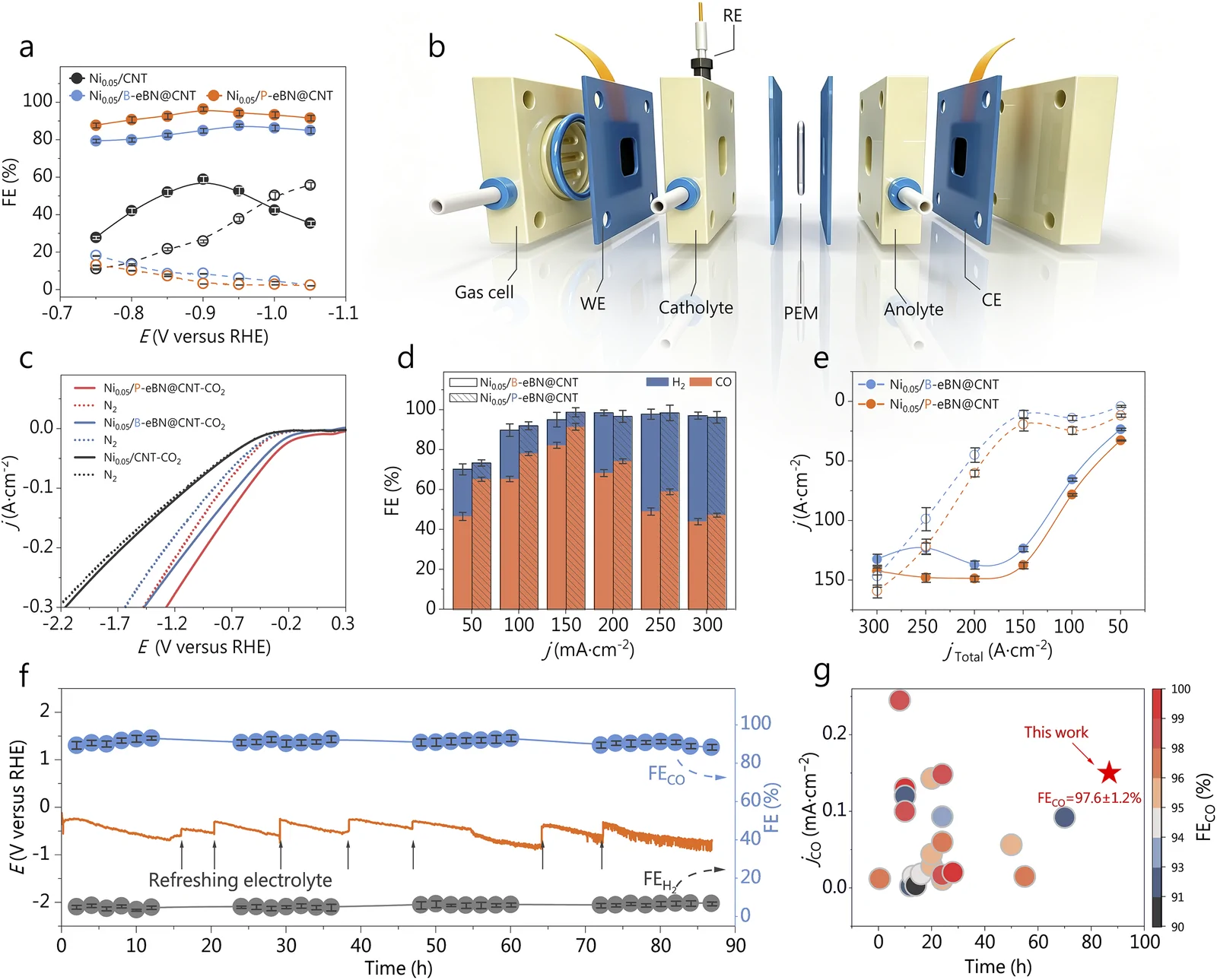
Electrocatalytic CO_2_RR performance. (a) Faradaic efficiencies of Ni_0.05_/P-eBN@CNT, Ni_0.05_/B-eBN@CNT and Ni_0.05_/CNT tested in an H-cell. Solid lines correspond to CO, while dashed lines stand for H_2_. (b) Structural sketch of the employed GDE flow cell setup. (c) LSV curves of Ni_0.05_/P-eBN@CNT, Ni_0.05_/B-eBN@CNT and Ni_0.05_/CNT tested in the flow cell. (d) Faradaic efficiencies of Ni_0.05_/P-eBN@CNT and Ni_0.05_/B-eBN@CNT tested in the flow cell. (e) *j*_CO_ of Ni_0.05_/P-eBN@CNT and Ni_0.05_/B-eBN@CNT tested in the flow cell. Solid lines correspond to CO, while dashed lines stand for H_2_. (f) Long-term chronopotentiometry profile together with matched product FE for Ni_0.05_/P-eBN@CNT at 150 mA cm^−2^ current density. (g) Comparison of electrocatalytic CO_2_ reduction performance among previously reported advanced Ni-based catalysts, where each data point corresponds to one catalyst specimen (detailed data are summarized in Table S4). Error bars represent standard deviations acquired from three separate replicate measurements.

Inspired by the prominent performance of Ni-based catalysts, Fe_0.05_/P-eBN@CNT and Co_0.05_/P-eBN@CNT were fabricated *via* an identical method, which confines Fe and Co species onto P-eBN@CNT, aiming to evaluate the universal applicability of this defective BN support. Multiple characterization studies were implemented to confirm the successful synthesis of the Fe- and Co-based counterparts. PXRD patterns (Fig. S13) of Fe_0.05_/P-eBN@CNT and Co_0.05_/P-eBN@CNT displayed no distinct diffraction peaks assigned to metallic crystalline phases, demonstrating the absence of aggregated metal nanoparticles. XPS measurements were further carried out to analyze the chemical environments of the two catalysts. The B 1s, C 1s and N 1s spectra show peak distributions consistent with those of Ni_0.05_/P-eBN@CNT, and characteristic B–C, B–O, C–N and N–B bonds are identified in both samples (Fig. S14 and S15). Subsequently, electrochemical CO_2_RR measurements were carried out under the identical conditions as those for the Ni-based catalysts. LSV curves confirm that both Fe_0.05_/P-eBN@CNT and Co_0.05_/P-eBN@CNT show notable CO_2_RR activity (Fig. S16). They retained FE_CO_ over 90% across a wide potential window (Fig. S17), with a maximum *j*_CO_ of 17.4 ± 0.4 mA cm^−2^ (Fe_0.05_/P-eBN@CNT) and 16.1 ± 0.4 mA cm^−2^ (Co_0.05_/P-eBN@CNT) at −1.05 V *versus* RHE (Fig. S18). EIS tests revealed low charge-transfer resistance, while *C*_dl_ measurements demonstrated large electrochemically active surface areas for both samples (Fig. S19–S21). The favourable performance delivered by both Fe_0.05_/P-eBN@CNT and Co_0.05_/P-eBN@CNT collectively demonstrates that this support also delivers promising CO_2_RR activity for Fe- and Co-based catalysts. Ni_0.05_/P-eBN@CNT with the best activity was selected for a 50-hour long-term durability test at an optimal potential of −0.9 V *versus* RHE (Fig. S22). Chronoamperometry results indicated only minor degradation of current density and FE_CO_ throughout the electrolysis process, demonstrating excellent catalytic durability.

To further verify the structural stability of the catalysts under operating conditions, systematic characterization studies were implemented for post-electrolysis samples. Post-reaction PXRD results reveal that Ni_0.05_/P-eBN@CNT still exhibits the characteristic (002) and (100) diffraction peaks of BN, with no detectable signals corresponding to metallic Ni^0^. In sharp contrast, distinct diffraction peaks assigned to metallic Ni^0^ emerge for the post-test Ni_0.05_/CNT (Fig. S23). This phenomenon demonstrates that Ni species without confinement by BN networks are prone to agglomeration under working conditions. The TEM image shows that the tubular structure of CNTs and the thin BN coating layer are well-preserved, without aggregation of metallic Ni nanoparticles observed (Fig. S24). AC-HAADF-STEM images coupled with elemental mapping further visualized discrete Ni single-atom bright spots, with homogeneous distribution of C, B, N, P and Ni elements without local metal enrichment (Fig. S25 and S26). Minor shifts in binding energy were detected for C 1s, B 1s and N 1s high-resolution XPS spectra, without new peaks originating from metal aggregation or leaching (Fig. S27). The consistent evidence from all post-catalysis characterization studies collectively reveals that the defective BN network can firmly anchor Ni single atoms. The support skeleton remains intact during long electrolysis, and Ni active sites are free from irreversible deactivation caused by aggregation or metal leaching, which accounts for the outstanding long-term catalytic stability of the catalyst.

Based on the intrinsic catalytic trends obtained from H-cell measurements, a flow cell equipped with a gas diffusion electrode (GDE) was further employed to conduct high-current amplification tests under industrially relevant mass-transport conditions (Fig. 3b). As presented in Fig. 3c, Ni_0.05_/P-eBN@CNT still delivers the optimal CO_2_RR activity. At a current density of 150 mA cm^−2^, its FE_CO_ reaches 91.5 ± 1.7%, which is superior to that of Ni_0.05_/B-eBN@CNT (Fig. 3d). The peak *j*_CO_ is 148.6 ± 2.3 mA cm^−2^, which is also higher than that of Ni_0.05_/B-eBN@CNT (136.8 ± 3.4 mA cm^−2^) (Fig. 3e). To assess the long-term operational stability at high-current density, an 87-hour durability measurement of Ni_0.05_/P-eBN@CNT was performed at 150 mA cm^−2^, where negligible decay of CO selectivity was observed (Fig. 3f). In contrast, Ni_0.05_/CNT suffers from severe decay in CO selectivity during a 43-hour stability test, which highlights the excellent stabilizing effect of the boron nitride network toward Ni single atoms (Fig. S28). For further evaluation, benchmarking against documented Ni SACs was carried out (Fig. 3g), demonstrating that the sustainable CO selectivity of Ni_0.05_/P-eBN@CNT exhibits competitive advantages among analogous materials.

### Insights into the CO_2_-to-CO mechanism of the CO_2_RR

To elucidate the intrinsic CO-producing mechanism of the CO_2_RR over defective BN-supported Ni, potential-dependent *operando* attenuated-total-reflection surface enhanced infrared adsorption spectroscopy (ATR-SEIRAS) was utilized to dynamically capture reactive intermediates, with the applied potential ranging from −0.2 to −1.4 V *versus* RHE (Fig. S29). As displayed in Fig. 4a, three characteristic absorption peaks located at 1288 cm^−1^, 1386 cm^−1^ and 1660 cm^−1^ are assigned to the symmetric and asymmetric stretching vibrations of the key *COOH intermediate, respectively.^54^ This observation indicates that the CO_2_-to-CO conversion should follow a two-step proton-coupled electron transfer pathway: CO_2_ molecules are initially anchored on Ni active sites *via* carbon terminal adsorption, followed by the first proton-coupled electron transfer to generate the *COOH intermediate. Subsequently, the *COOH species undergoes another proton-coupled electron transfer and desorbs to release the CO product (Fig. 4b).^55^ Remarkably, the absorption peak intensity of *COOH for Ni_0.05_/P-eBN@CNT is obviously stronger than that of Ni_0.05_/B-eBN@CNT (Fig. S30). To further clarify the catalytic discrepancies induced by different acid-etched supports at the atomic level, DFT calculations were performed based on the constructed models abbreviated as Ni-NP (Fig. S31), Ni-SA-eBN (Fig. S32), and Ni-SA-P-eBN (Fig. S33), corresponding to the three catalysts of Ni_0.05_/P-eBN@CNT, Ni_0.05_/B-eBN@CNT and Ni_0.05_/CNT, respectively. We also attempted to anchor B(OH)_4_^−^ on defective eBN substrates during model construction. However, structural optimization revealed that adsorbed B(OH)_4_^−^ spontaneously decomposed, suggesting poor surface stability. In contrast, phosphate species could remain stably anchored on the support.

**Fig. 4:**
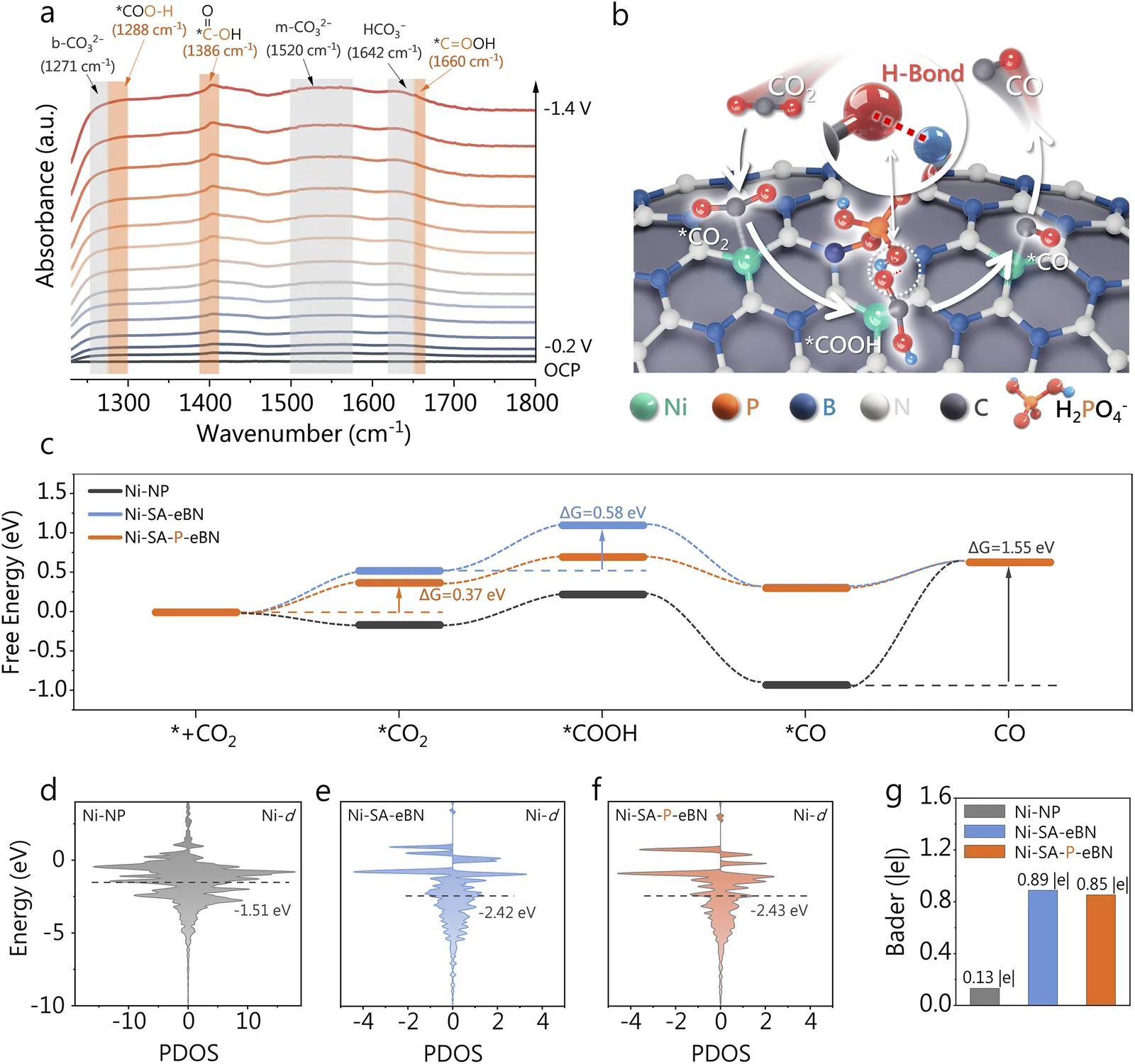
Electrocatalytic mechanism of CO_2_-to-CO conversion. (a) Potential-dependent *operando* ATR-SEIRAS spectra of Ni_0.05_/P-eBN@CNT from −0.2 to −1.4 V *versus* RHE. (b) Schematic sketch depicting the CO_2_-to-CO transformation route enabled by hydrogen-bond mediated stabilization for the *COOH intermediate. (c) Free energy profiles of Ni-NP, Ni-SA-eBN and Ni-SA-P-eBN for the CO_2_RR. PDOS diagrams of Ni d-orbitals for (d) Ni-NP, (e) Ni-SA-eBN and (f) Ni-SA-P-eBN, with corresponding d-band centers marked. (g) Bader charge calculation for surface Ni sites over Ni-NP, Ni-SA-eBN and Ni-SA-P-eBN.

Free energy calculations demonstrate that the Ni sites in Ni-NP possess high electron density, which facilitates π-back-donation and strengthens the adsorption of *CO. Consequently, the free energy change (Δ*G*) for the *CO desorption step reaches up to 1.55 eV, severely hindering continuous CO_2_RR catalysis. Ni-SA-eBN and Ni-SA-P-eBN exhibit comparable electron density at active sites, leading to similar free energy barriers for *CO desorption. Nevertheless, phosphorus-containing moieties on Ni-SA-P-eBN may stabilize the critical *COOH intermediate *via* intermolecular hydrogen bonds, which likely contributes to the observed kinetic advantages (Fig. 4c).^56^ The rate-determining step (RDS) of Ni-SA-eBN is the formation of *COOH with a Δ*G* of 0.58 eV. Benefiting from hydrogen-bond stabilization, the energy change for *COOH generation on Ni-SA-P-eBN is reduced to 0.32 eV (Table S3), and the RDS shifts to the CO_2_ adsorption with Δ*G* = 0.37 eV.^57^ The Ni d-band centers extracted from PDOS profiles (Fig. 4d–f) further rationalize the distinct adsorption behaviors of reaction intermediates. The d-band center of Ni-NP is located at −1.51 eV, much closer to the Fermi level. Such an upward-shifted d-band center enhances orbital overlap between Ni sites and *CO, which promotes π-back-donation and results in excessively strong *CO binding,^58,59^ consistent with the high energy barrier for *CO desorption observed in free energy diagrams. In contrast, the d-band centers of Ni-SA-eBN (−2.42 eV) and Ni-SA-P-eBN (−2.43 eV) shift markedly downward away from the Fermi level, effectively weakening the *CO adsorption and facilitating its desorption.^60^ The slightly downshifted d-band center of Ni-SA-P-eBN relative to Ni-SA-eBN enables moderate tuning of intermediate binding strength. Bader charge analysis (Fig. 4g) further corroborates this electronic regulation: Ni-NP only exhibits a Bader charge of 0.13|*e*|, while Ni-SA-eBN (0.89|*e*|) and Ni-SA-P-eBN (0.85|*e*|) display markedly higher positive charge accumulation on Ni sites, verifying more severe electron transfer from Ni to the defective BN support. Collectively, the single-atom Ni supported on phosphoric acid-etched BN networks enables hydrogen-bond-mediated stabilization of adsorbed *COOH on Ni, thereby accelerating the production of CO.

## Conclusions

In summary, we propose a defective BN support *via* a phosphoric-acid etching strategy to stabilize Ni single atoms, which also serves as an effective support for Co and Fe materials. The optimal Ni based catalyst Ni_0.05_/P-eBN@CNT exhibits a CO selectivity as high as 97.6 ± 1.2% at a current density of 150 mA cm^−2^ in a flow cell and retained sustainable stability. On one hand, the defective BN support can effectively modulate the electronic structure of the Ni sites, thereby optimizing the adsorption/desorption of intermediates; on the other hand, it can also stabilize the metal single atoms, preventing their agglomeration during electrocatalysis. In addition, *operando* ATR-SEIRAS and DFT calculations reveal that the modified phosphate ions may stabilize *COOH through hydrogen bonding, thereby lowering the reaction energy barrier. This study provides a viable surface modification strategy for BN-based single-atom catalysts, deepens the mechanism understanding of molecule-mediated CO_2_ electroreduction, and contributes to the design of high-performance electrocatalysts for the practical conversion of CO_2_ to CO.

## Author contributions

C.-T. H. L.-M. C. and H.-H. H. conceived the idea, designed the study, and guided the project. X. L., Y.-T. W. and W.-Z. D. conducted material design, synthesis, and characterization. X. L., H.-H. H. and Y.-T. X. performed theoretical calculations. X. L., Y.-T. W., W.-Z. D., Q.-R. T., Z.-X. W., Y.-J. S., H.-M. L., and W.-J. H. conceived the electrocatalytic experiments and performed the corresponding data analysis. X. L., H.-H. H., L.-M. C., and C.-T. H. drafted the manuscript. All authors discussed the results and commented on the manuscript.

## Conflicts of interest

There are no conflicts to declare.

## Supplementary Material

SC-OLF-D6SC06494A-s001

## Data Availability

All data supporting this study are available within the article and its supplementary information (SI). The SI contains the experimental section and supplemental data for catalyst characterization and electrocatalytic performances. Supplementary information is available. See DOI: https://doi.org/10.1039/d6sc06494a.
