# Supplementary material for: Phosphoric acid-etched boron nitride as a single-atom support for boosting CO2 electroreduction at high current density
Source: Chem Sci. 2026 Oct 12. Online ahead of print. doi: 10.1039/d6sc06494a (PMC13678589; doi:10.1039/d6sc06494a)
Supplement: Supplementary file 1 [file SC-OLF-D6SC06494A-s001.pdf]

Supplementary Information (SI) for Chemical Science.
This journal is © The Royal Society of Chemistry

# Supplementary Information

## Phosphoric acid-etched boron nitride as a single-atom support for boosting $CO_2$ electroreduction at high current density

*Xuan Li*,†[a] *Yu-Ting Wang*,†[a] *Wei-Zhen Deng*,†[a] *Qian-Run Tan*,[a] *Hong-Mei Liu*,[a] *Wen-Juan Huang*,[a] *Li-Dong Wang*,[a] *Zi-Xuan Wu*,[a] *Yuan-Jing Suo*,[a] *Yan-Tong Xu*,[b] *Li-Ming Cao*,*[a] *Hai-Hua Huang*,*[a] *Chun-Ting He*[*a]

[a]Key Lab of Fluorine and Silicon for Energy Materials and Chemistry of Ministry of Education, College of Chemistry and Materials, Jiangxi Normal University, Nanchang 330022, China

[b]School of Advanced Energy, Sun Yat-Sen University (Shenzhen), Shenzhen 518107, China

[*]Corresponding author.

*E-mail address:* huanghh@jxnu.edu.cn (H. H. Huang); caolm@jxnu.edu.cn (L. M. Cao); hct@jxnu.edu.cn (C. T. He)

# Table of Contents

Experimental Section ........ S4
Fig. S1. SEM and TEM images of BN@CNT ........ S8
Fig. S2. Raman patterns of BN@CNT and Peak-fitting plot for BN $E_{2g}$ ........ S9
Fig. S3. FTIR spectra of $Ni_{0.05}$/CNT, $Ni_{0.05}$/B-eBN@CNT and $Ni_{0.05}$/P-eBN@CNT ........ S10
Fig. S4. PXRD patterns of P-eBN@CNT, B-eBN@CNT and BN@CNT ........ S11
Fig. S5. High-resolution XPS spectra of B 1*s* of $Ni_{0.05}$/P-eBN@CNT and $Ni_{0.05}$/B-eBN@CNT ........ S12
Fig. S6. High-resolution XPS spectra of N 1*s* of $Ni_{0.05}$/P-eBN@CNT and $Ni_{0.05}$/B-eBN@CNT ........ S13
Fig. S7. FE of $Ni_{0.03}$/B-eBN@CNT, $Ni_{0.05}$/B-eBN@CNT and $Ni_{0.05}$/CNT tested in H-cell ........ S14
Fig. S8. LSV curves of $Ni_{0.05}$/P-eBN@CNT, $Ni_{0.05}$/B-eBN@CNT, $Ni_{0.05}$/CNT and P-eBN@CNT tested in H-cell ........ S15
Fig. S9. $j_{CO}$ of $Ni_{0.05}$/P-eBN@CNT, $Ni_{0.05}$/B-eBN@CNT and $Ni_{0.05}$/CNT tested in H-cell ........ S16
Fig. S10. CVs of P-eBN@CNT, $Ni_{0.05}$/CNT, $Ni_{0.05}$/B-eBN@CNT and $Ni_{0.05}$/P-eBN@CNT in 0.5 M $KHCO_3$ solution at different scan rates ........ S17
Fig. S11. $C_{dl}$ plots of P-eBN@CNT, $Ni_{0.05}$/CNT, $Ni_{0.05}$/B-eBN@CNT and $Ni_{0.05}$/P-eBN@CNT in H-cell ........ S18
Fig. S12. Nyquist plot curves of P-eBN@CNT, $Ni_{0.05}$/CNT, $Ni_{0.05}$/B-eBN@CNT and $Ni_{0.05}$/P-eBN@CNT in H-cell ........ S19
Fig. S13. PXRD patterns of $Ni_{0.05}$/P-eBN@CNT, $Fe_{0.05}$/P-eBN@CNT and $Co_{0.05}$/P-eBN@CNT ........ S20
Fig. S14. High-resolution XPS spectra of $Fe_{0.05}$/P-eBN@CNT of C 1*s*, N 1*s* and B 1*s* ........ S21
Fig. S15. High-resolution XPS spectra of $Co_{0.05}$/P-eBN@CNT of C 1*s*, N 1*s* and B 1*s* ........ S22
Fig. S16. LSV curves of $Ni_{0.05}$/P-eBN@CNT, $Fe_{0.05}$/P-eBN@CNT, $Co_{0.05}$/P-eBN@CNT and $Ni_{0.05}$/CNT tested in H-cell ........ S23
Fig. S17. FE of $Ni_{0.05}$/P-eBN@CNT, $Fe_{0.05}$/P-eBN@CNT and $Co_{0.05}$/P-eBN@CNT tested in H-cell ........ S24
Fig. S18. $j_{CO}$ of $Ni_{0.05}$/P-eBN@CNT, $Fe_{0.05}$/P-eBN@CNT and$Co_{0.05}$/P-eBN@CNT tested in H-cell ........ S25
Fig. S19. CVs of $Fe_{0.05}$/P-eBN@CNT and $Co_{0.05}$/ CNT in 0.5 M $KHCO_3$ solution at different scan rates ........ S26
Fig. S20. $C_{dl}$ plots of $Ni_{0.05}$/P-eBN@CNT, $Fe_{0.05}$/P-eBN@CNT and $Co_{0.05}$/P-eBN@CNT in H-cell ........ S27
Fig. S21. Nyquist plot curves of $Ni_{0.05}$/P-eBN@CNT, $Fe_{0.05}$/P-eBN@CNT and $Co_{0.05}$/P-eBN@CNT in H-cell ........ S28
Fig. S22. Long-term potentiostatic test and the corresponding formate Faraday efficiencies of $Ni_{0.05}$/P-eBN@CNT at −0.90 V vs. RHE ........ S29
Fig. S23. PXRD patterns of $Ni_{0.05}$/P-eBN@CNT, $Ni_{0.05}$/P-eBN@CNT-After $CO_2$RR and $Ni_{0.05}$/CNT-After $CO_2$RR ........ S30
Fig. S24. TEM image of $Ni_{0.05}$/P-eBN@CNT-After $CO_2$RR ........ S31
Fig. S25. AC-HAADF-STEM image of $Ni_{0.05}$/P-eBN@CNT-After $CO_2$RR ........ S32

Fig. S26. EDX elemental mapping images of $Ni_{0.05}$/P-eBN@CNT-After $CO_2$RR ........................S33
Fig. S27. High-resolution XPS spectra of $Ni_{0.05}$/P-eBN@CNT-After $CO_2$RR of C 1*s*, N 1*s* and B 1*s*. ........................S34
Fig. S28. Long-term chronopotentiometry profile test for $Ni_{0.05}$/CNT ........................S35
Fig. S29. The photograph of the operando ATR-SEIRAS system ........................S36
Fig. S30. Potential-dependent *operando* ATR-SEIRAS spectra of $Ni_{0.05}$/B-eBN@CNT from −0.2 to −1.4 V vs. RHE ........................S37
Fig. S31. The intermediates and their structural models involved in the electrochemical reduction of $CO_2$ to CO on Ni-NP ........................S38
Fig. S32. The intermediates and their structural models involved in the electrochemical reduction of $CO_2$ to CO on Ni-SA-eBN........................S39
Fig. S33. Pathway and structural modeling of $CO_2$RR to CO on Ni-SA-P-eBN ........................S40
Table S1. ICP-OES data of $Ni_{0.05}$/P-eBN@CNT, $Ni_{0.05}$/B-eBN@CNT and $Ni_{0.05}$/CNT ........................S41
Table S2. EXAFS fitting parameters at the Ni *K*-edge for various samples ........................S42
Table S3. Relative Gibbs free energies (eV) of $CO_2$RR intermediates over Ni-SA-P-eBN, Ni-SA-B-eBN and $Ni^0$-NP........................S43
Table S4. Comparison of the $CO_2$RR performance of Ni-based catalysts ........................S44
References........................S46

## Experimental Section

### Materials

Nickel nitrate hexahydrate ($Ni(NO_3)_2 \cdot 6H_2O$, 99.0%), iron nitrate nonahydrate ($Fe(NO_3)_2 \cdot 9H_2O$, 98.0%), cobalt nitrate hexahydrate ($Co(NO_3)_2 \cdot 6H_2O$, AR), urea ($CO(NH_2)_2$, 99.0%), boric acid ($H_3BO_3$, 99.0%), phosphoric acid ($H_3PO_4$, AR), isopropyl alcohol (99.9%), potassium bicarbonate ($KHCO_3$, 99.5%), anhydrous ethanol (99.99%) and 5 wt% Nafion solution were purchased from Shanghai Aladdin Biochemical Technology Co., Ltd. Amino-functionalized multi-walled carbon nanotubes (CNT, 95.0%) were purchased from Beijing Innochem Technology Co., Ltd. Proton exchange membrane (10 cm × 10 cm, Nafion series) was obtained from DuPont, USA. For all experiments, an ultrapure water purifier with a resistivity of not less than 18 MΩ·cm was used. The purity of the $N_2$ and $CO_2$ used was 99.99%. All chemicals and materials were used without further purification.

### Catalyst Synthesis

**Synthesis of BN@CNT**

Urea (1.186 g), boric acid (0.244 g) and amino-modified multi-walled carbon nanotubes (0.50 g) were fully ground in an agate mortar for 20 min to obtain a uniform black powder mixture. The mixed powder was transferred into an alumina crucible and placed in a tube furnace. Under an $Ar/NH_3$ mixed atmosphere, the furnace was heated to 1050℃ at a heating rate of 15 ℃ $min^{-1}$ and held at this temperature for 2 h. After cooling naturally to room temperature, the black composite powder denoted as BN@CNT was collected.

**Synthesis of B-eBN@CNT and P-eBN@CNT**

500 mg of as-prepared BN@CNT powder was dispersed in 0.5 M aqueous boric acid solution. The suspension was heated to 90℃ under oil bath stirring for 30 min. Afterwards, the solid precipitate was collected by suction filtration and repeatedly rinsed with deionized water. The obtained solid was vacuum-dried at 60℃ overnight

to yield boric acid-etched BN@CNT (B-eBN@CNT). The preparation of P-eBN@CNT followed the same procedure as B-eBN@CNT, with only 0.5 M boric acid substituted by an equal volume of 0.5 M phosphoric acid.

**Synthesis of $Ni_{0.03}$/B-eBN@CNT, $Ni_{0.05}$/B-eBN@CNT and $Ni_{0.1}$/B-eBN@CNT**

100 mg of B-eBN@CNT support was dispersed in 5 mL deionized water with vigorous stirring to form a homogeneous suspension. Three batches of nickel precursor solutions were prepared by adding 51.5 μL, 85.2 μL and 170.4 μL of 0.01 M nickel nitrate hexahydrate aqueous solution into the above suspensions, respectively. Each mixture was continuously stirred for 5 h to achieve uniform adsorption of $Ni^{2+}$ ions on the B-eBN@CNT substrate, followed by freeze-drying for 24 h to completely remove solvent. The dried solid powders were calcined under the same annealing parameters as BN@CNT (1050℃, 2 h under Ar/$NH_3$ atmosphere) to obtain black catalysts, which were named $Ni_{0.03}$/B-eBN@CNT, $Ni_{0.05}$/B-eBN@CNT and $Ni_{0.1}$/B-eBN@CNT in accordance with the feeding Ni dosage.

**Synthesis of $Ni_{0.05}$/P-eBN@CNT, $Fe_{0.05}$/P-eBN@CNT and $Co_{0.05}$/P-eBN@CNT**

The preparation routes of $Ni_{0.05}$/P-eBN@CNT, $Fe_{0.05}$/P-eBN@CNT and $Co_{0.05}$/P-eBN@CNT adopted the identical experimental operations and parameters described for $Ni_{0.05}$/B-eBN@CNT in Section 2.3, with the only difference that B-eBN@CNT support was substituted with P-eBN@CNT.

**Characterization**

Powder X-ray diffraction (PXRD) characterizations were carried out on a Rigaku MiniFlex600 powder diffractometer equipped with Cu Kα radiation source. Transmission electron microscopy (TEM, JEM2100F) and aberration-corrected high-angle annular dark-field scanning transmission electron microscopy (AC-HAADF-STEM) were adopted to analyze the morphological features of as-prepared catalysts. Inductively coupled plasma optical emission spectroscopy (ICP-OES, PerkinElmer 8300) was utilized to quantify the elemental loadings of Ni and P. An Escalab 250Xi X-ray photoelectron spectrometer was employed for X-ray photoelectron spectroscopy

(XPS) tests to probe the surface chemical composition of various samples. Synchrotron-based X-ray absorption fine structure (XAFS) experiments were implemented at the Spring-8 beamline facility in Japan. In-situ infrared spectroscopic measurements were conducted using a Thermo/Nicolet iS50 instrument coupled with a CHI-760E electrochemical workstation.

**Electrochemical Measurements**

All electrochemical measurements in the H-cell were performed using a standard three-electrode configuration on a CHI-760E workstation. The H-cell system comprised an Ag/AgCl reference electrode, a Pt foil counter electrode, and a glassy carbon working electrode (GCE, 5 mm in diameter), with the cathode and anode compartments separated by a proton exchange membrane. The electrochemical tests were performed in 0.5 M $KHCO_3$ aqueous electrolyte without *iR* compensation. For catalyst ink preparation, 4 mg of sample was homogenously dispersed in a mixture of 190 μL isopropanol and 10 μL Nafion solution via 30-minute sonication to achieve a uniform ink-like suspension. A catalyst loading of 1 $mg \cdot cm^{-2}$ was realized by pipetting 10 μL of catalyst ink onto a 1 × 1 $cm^2$ glassy carbon working electrode. Prior to electrochemical characterization, the electrolyte was purged with either $CO_2$ or $N_2$ gas at a constant flow rate of 20 sccm for 30 minutes using a calibrated mass flow controller to ensure complete gas saturation. The homogeneous dispersion was uniformly deposited via spray coating onto carbon paper, with a precisely aligned square mask defining the deposition area to fabricate a 1 × 1 $cm^2$ catalytic layer with a controlled catalyst loading of 1 $mg \cdot cm^{-2}$ that functioned as the gas diffusion electrode (GDE) within the flow cell configuration. Electrochemical measurements were performed in a custom-designed reactor featuring discrete cathode and anode chambers (1 cm × 1 cm × 1 cm), physically separated by a proton exchange membrane. Both compartments incorporated dedicated electrolyte inlet/outlet ports, with electrolyte circulation maintained at 5 sccm through precision peristaltic pumps. The three-electrode system incorporated an Ag/AgCl reference electrode positioned within the cathode chamber, while both the GDE and platinum counter electrode maintained identical geometric

surface areas (1 $cm^2$). $CO_2$RR experiments were systematically conducted under ambient conditions utilizing 1.0 M $KHCO_3$ electrolyte within the flow cell assembly. During electrochemical testing, continuous $CO_2$ saturation was achieved through controlled gas-phase delivery (5 sccm) to the cathodic GDE via dedicated gas channels, and no *iR* compensation was applied for all flow cell electrochemical measurements. All potentials were calibrated against the reversible hydrogen electrode: $E_{RHE} = E_{Ag/AgCl} + 0.059 \times pH$. The gas produced in the electrocatalytic reduction process were detected by gas chromatography (GC). The gas product Faraday efficiency is calculated as follows: FE(%) = $(n_g \times N \times F \times Flowrate \times P)/(R \times T \times I)$, where $n_g$ is the gas product content (ppm), *Flowrate* is the gas flow rate (sccm), *P* is the ambient pressure (101.325 kPa), *R* is the gas constant (8.314 $J \cdot K^{-1} \cdot mol^{-1}$), T is the ambient temperature (K) and *I* is the average current density ($mA \cdot cm^{-2}$).

**Computational Methods**

DFT computations were implemented within the Vienna Ab initio Simulation Package (VASP)[1-2]. The Perdew-Burke-Ernzerhof (PBE) functional was adopted to depict interactions between ionic cores and valence electrons, while the projector-augmented wave (PAW) approach was utilized to characterize electron-ion interactions[3-4]. An energy cutoff of 480 eV was adopted throughout all calculations. The convergence thresholds for total energy and atomic forces were separately defined as $10^{-5}$ eV and 0.03 eV $Å^{-1}$. The Brillouin zone was sampled using a 2 × 2 × 1 k-point mesh[5]. Structural models were built from boron nitride crystal with lattice dimensions of 15.060 Å × 15.060 Å, and a vacuum layer of approximately 15 Å was introduced to eliminate spurious interactions originating from periodic boundary conditions. Thermal corrections to the Gibbs free energy for all intermediates along the reaction pathway were evaluated via the VASPKIT code[6].

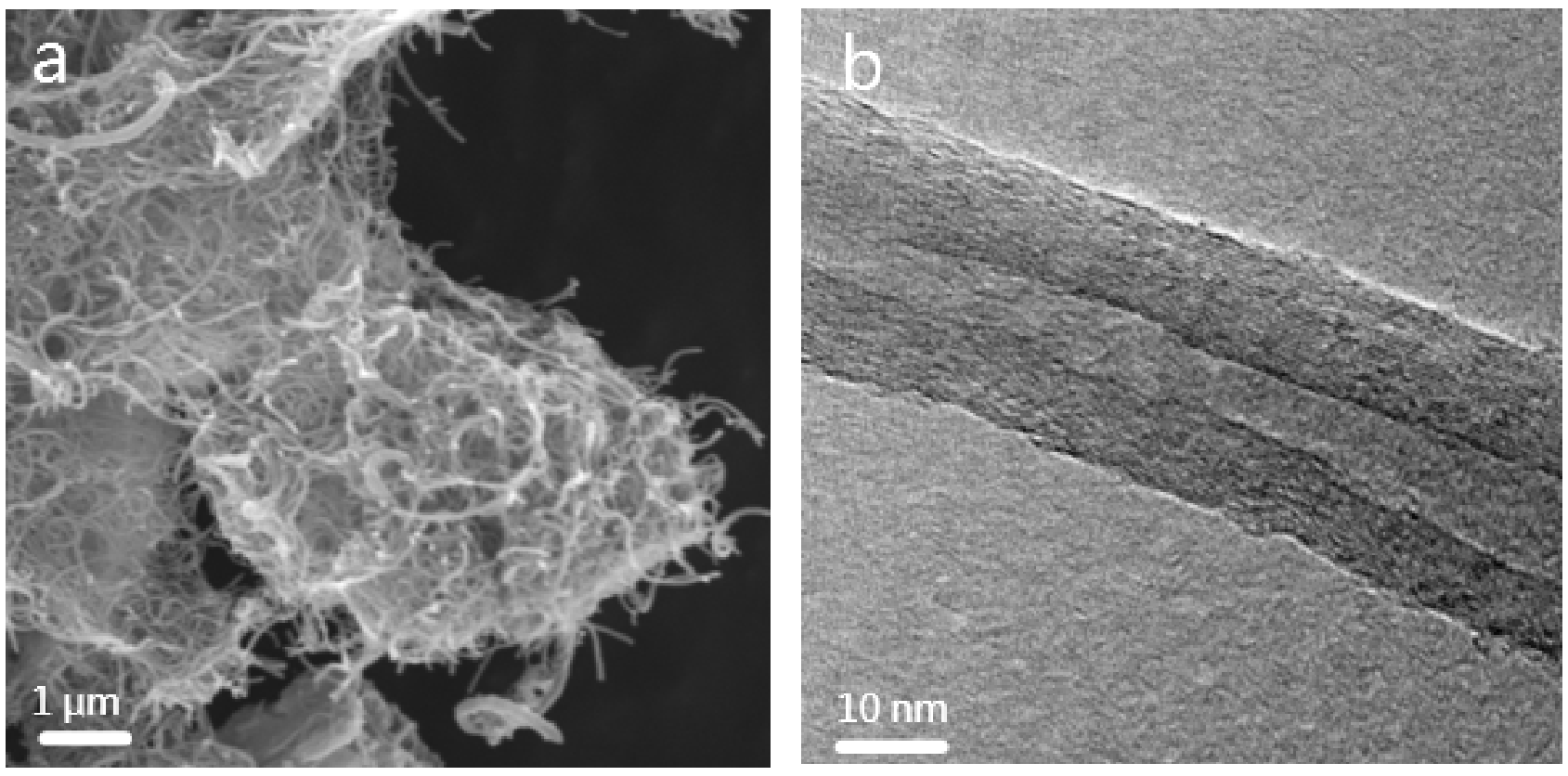


**Fig. S1.** (a) SEM image of BN@CNT. (b) TEM image of BN@CNT.

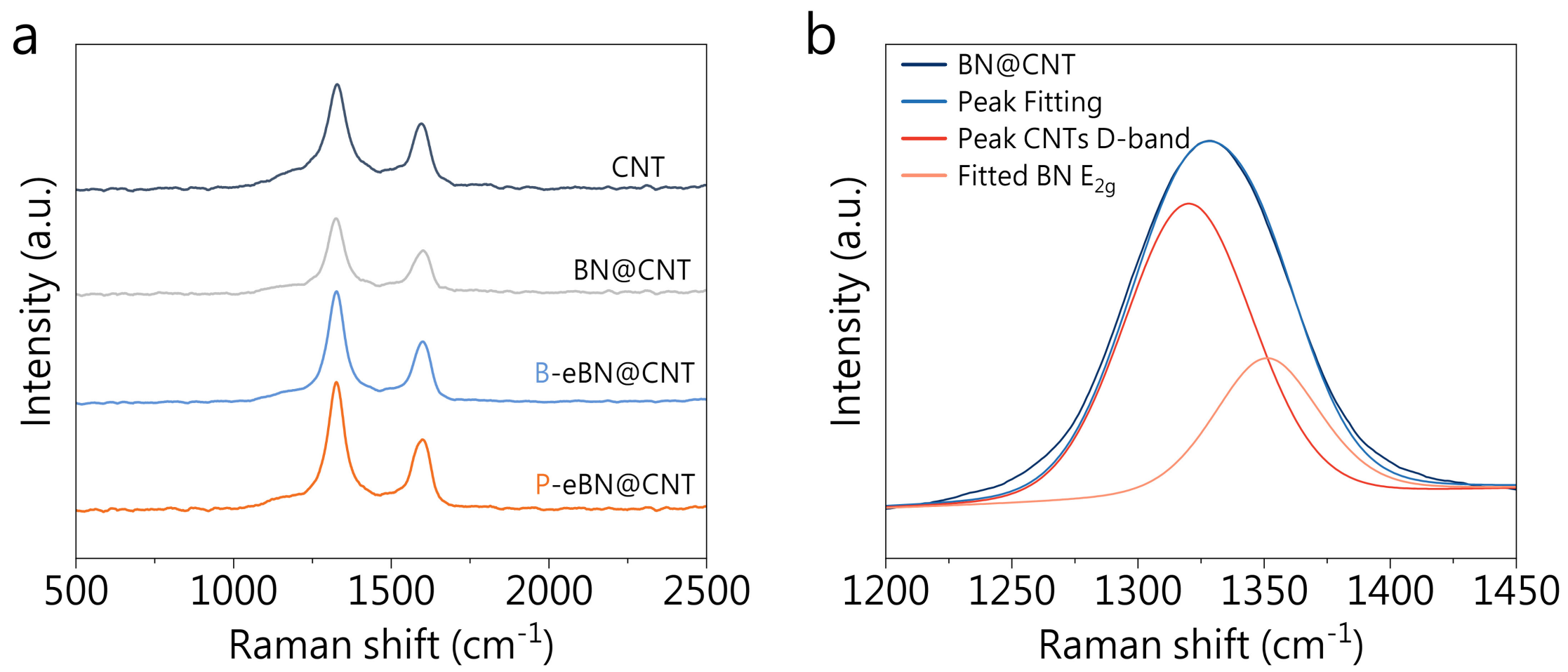


**Fig. S2.** (a) Raman patterns of CNT, BN@CNT, B-eBN@CNT and P-eBN@CNT. (b) Peak-fitting plot for BN $E_{2g}$.

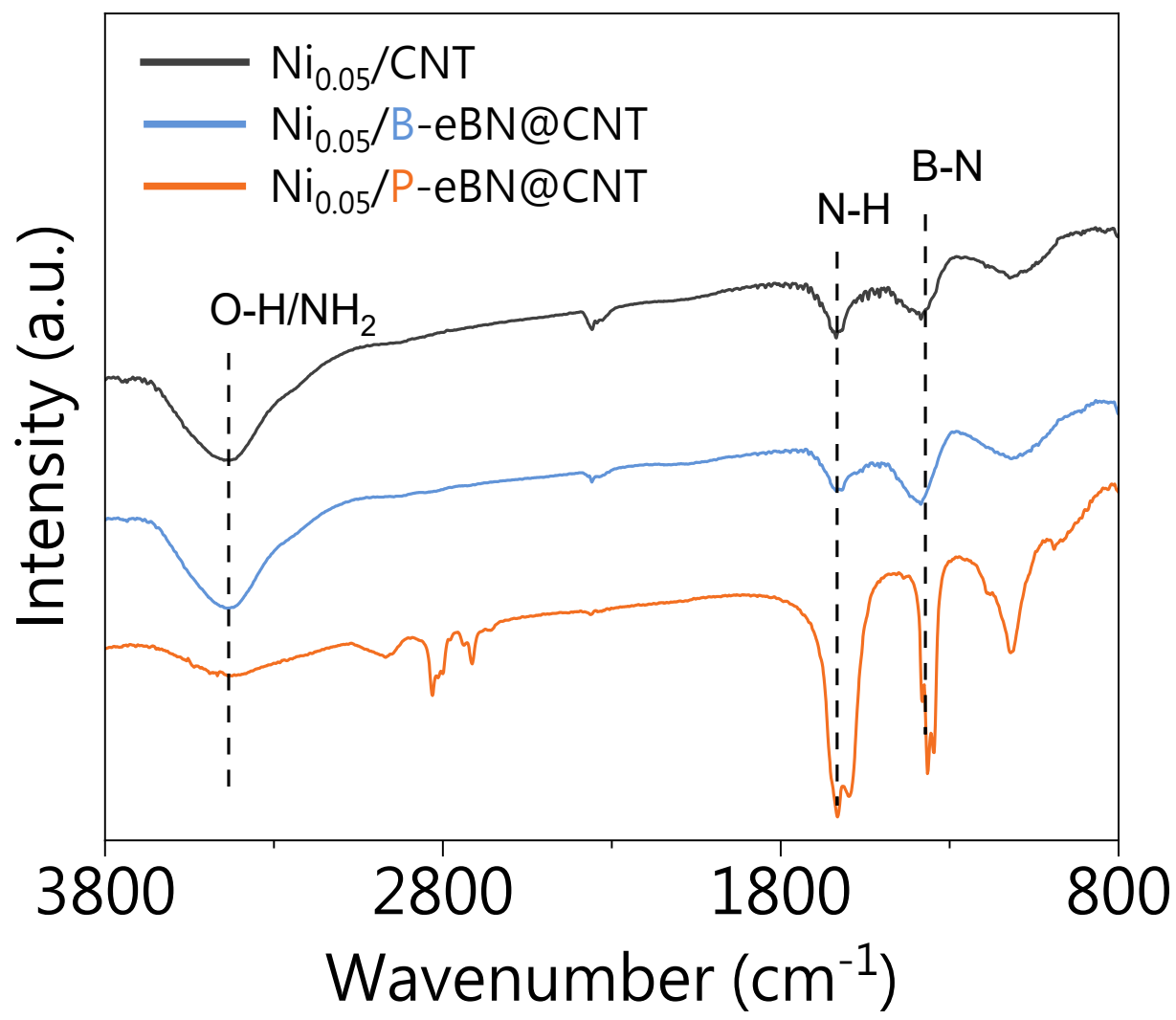


**Fig. S3.** FTIR spectra of $Ni_{0.05}$/CNT, $Ni_{0.05}$/B-eBN@CNT and $Ni_{0.05}$/P-eBN@CNT.

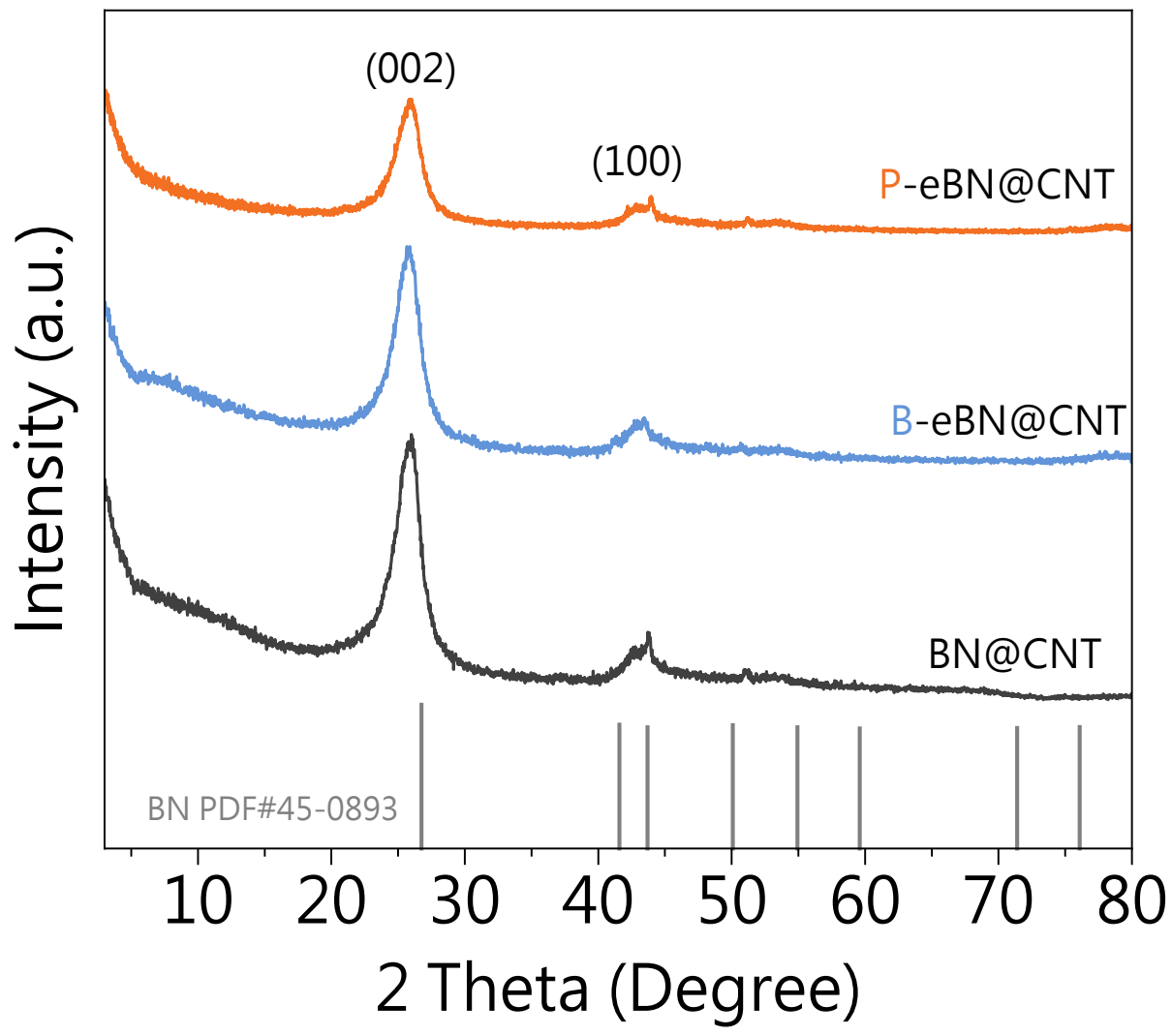


**Fig. S4.** PXRD patterns of P-eBN@CNT, B-eBN@CNT and BN@CNT.

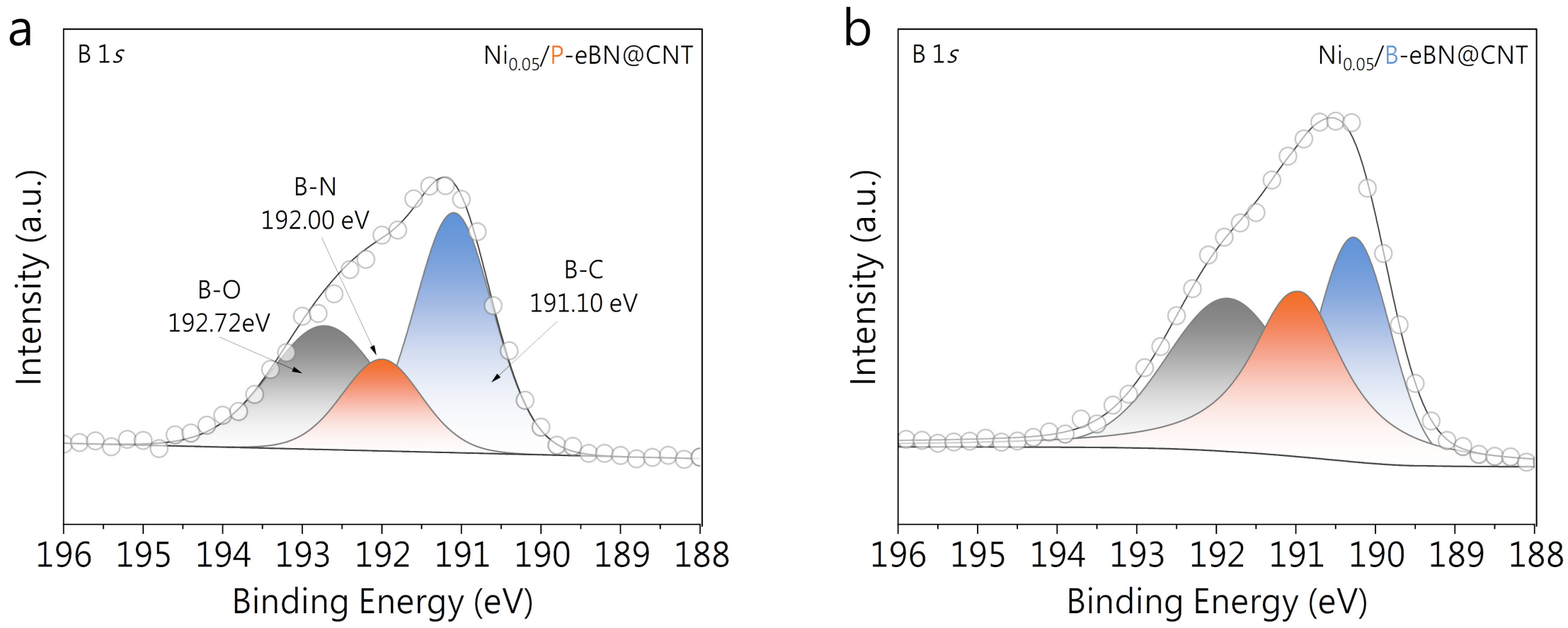


**Fig. S5.** High-resolution XPS spectra of B 1*s* of (a) $Ni_{0.05}$/P-eBN@CNT and (b) $Ni_{0.05}$/B-eBN@CNT

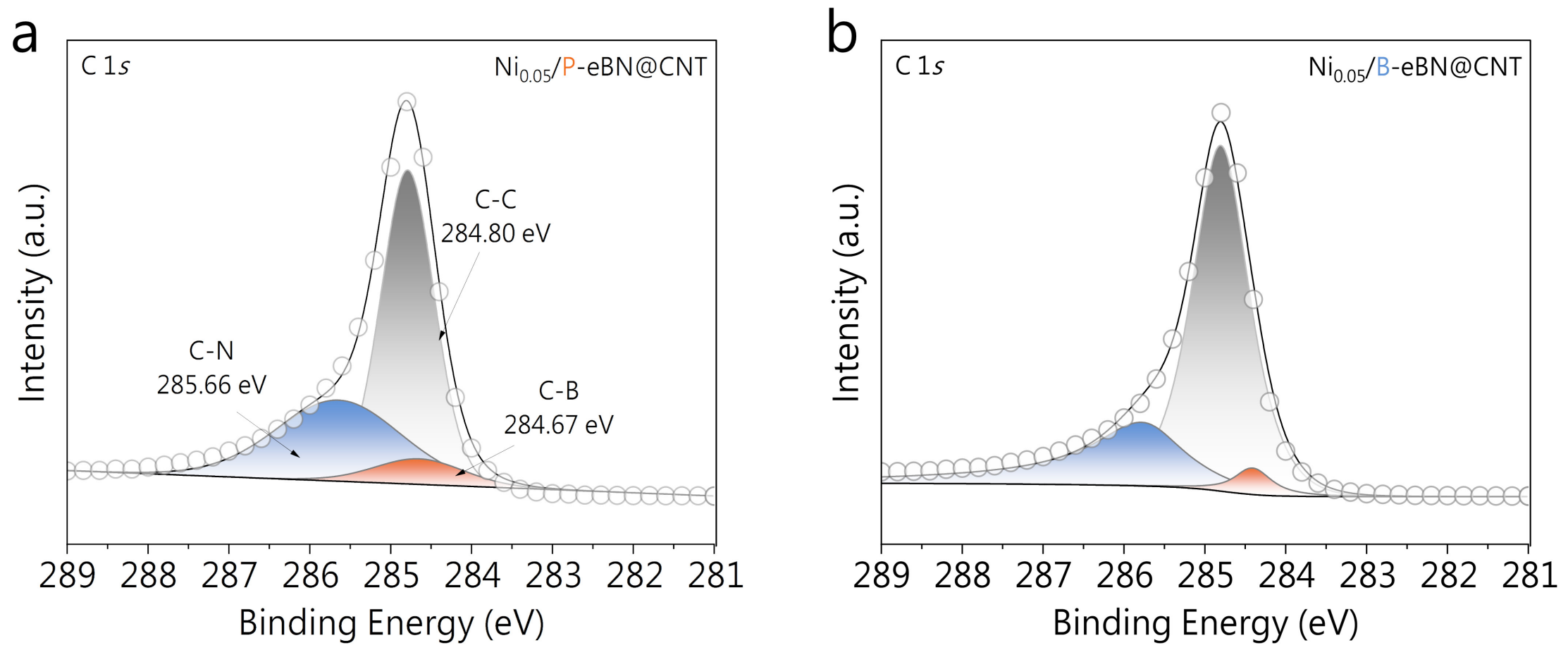


**Fig. S6.** High-resolution XPS spectra of C 1*s* of (a) $Ni_{0.05}$/P-eBN@CNT and (b) $Ni_{0.05}$/B-eBN@CNT.

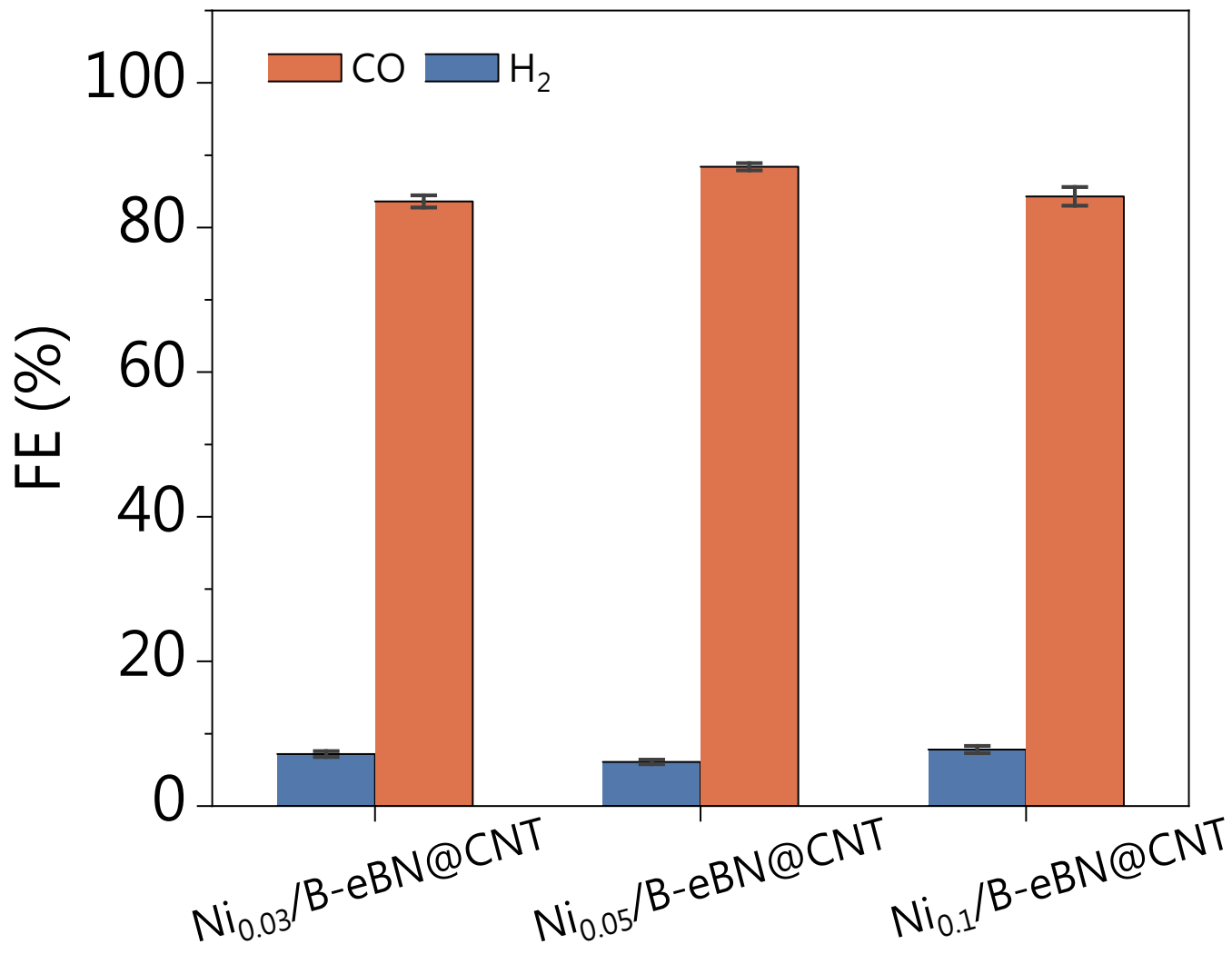


**Fig. S7.** Faradaic efficiencies of $Ni_{0.03}$/B-eBN@CNT, $Ni_{0.05}$/B-eBN@CNT and $Ni_{0.05}$/CNT tested in H-cell.

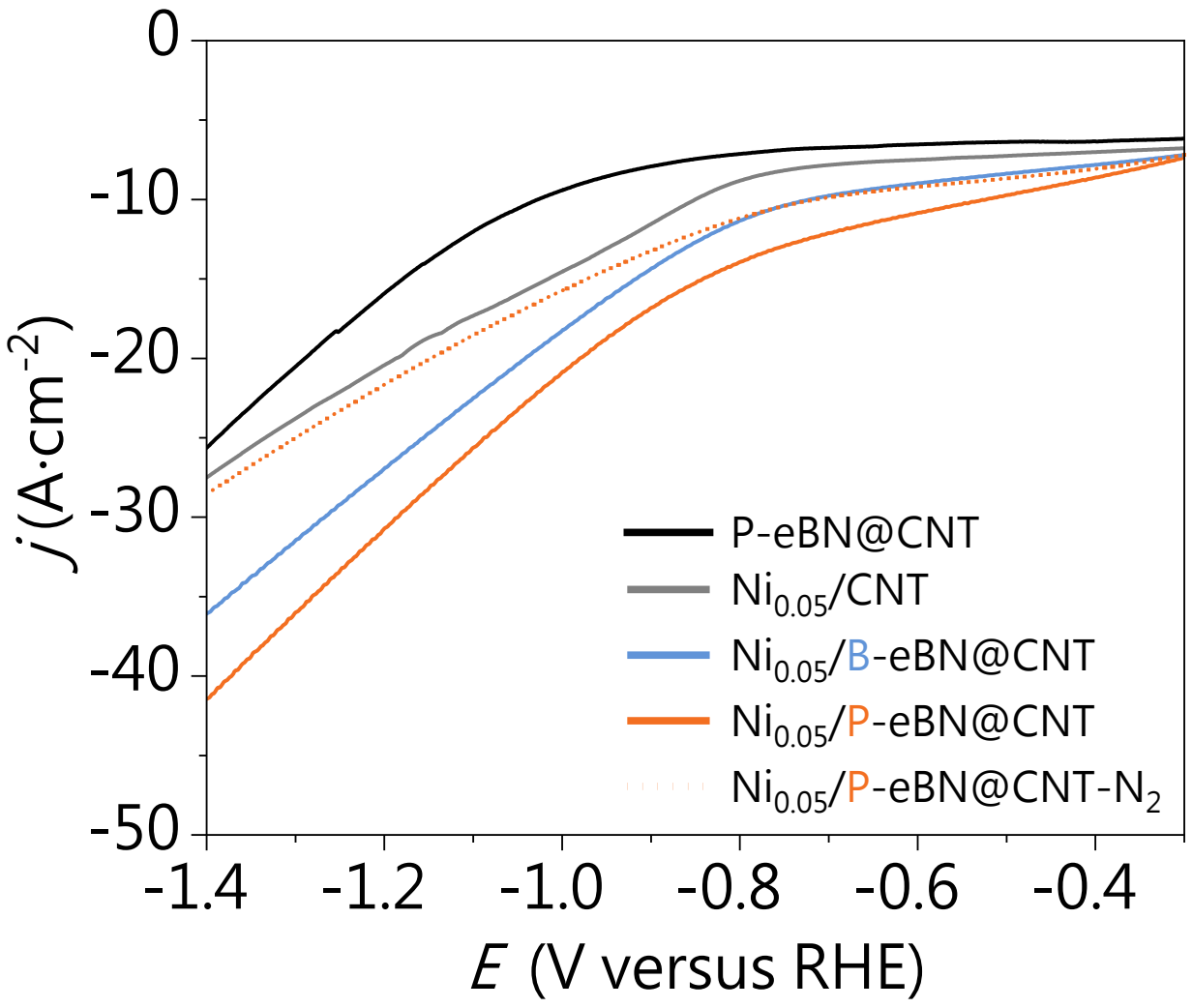


**Fig. S8.** LSV curves of $Ni_{0.05}$/P-eBN@CNT, $Ni_{0.05}$/B-eBN@CNT, $Ni_{0.05}$/CNT and P-eBN@CNT tested in H-cell. The dotted line represents the results of Test $Ni_{0.05}$/P-eBN@CNT conducted in a $N_2$ atmosphere.

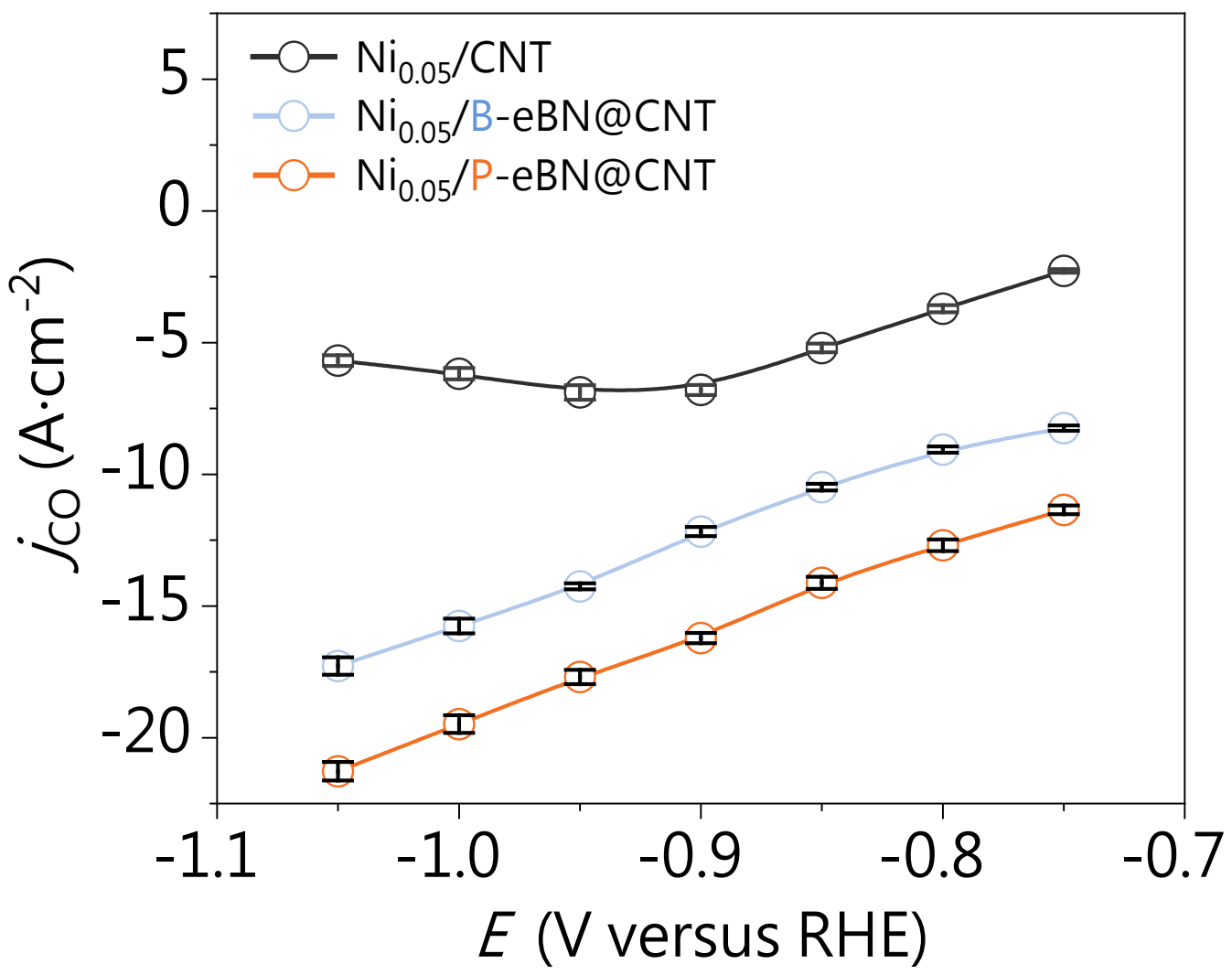


**Fig. S9.** $j_{CO}$ of $Ni_{0.05}$/P-eBN@CNT, $Ni_{0.05}$/B-eBN@CNT and $Ni_{0.05}$/CNT tested in H-cell.

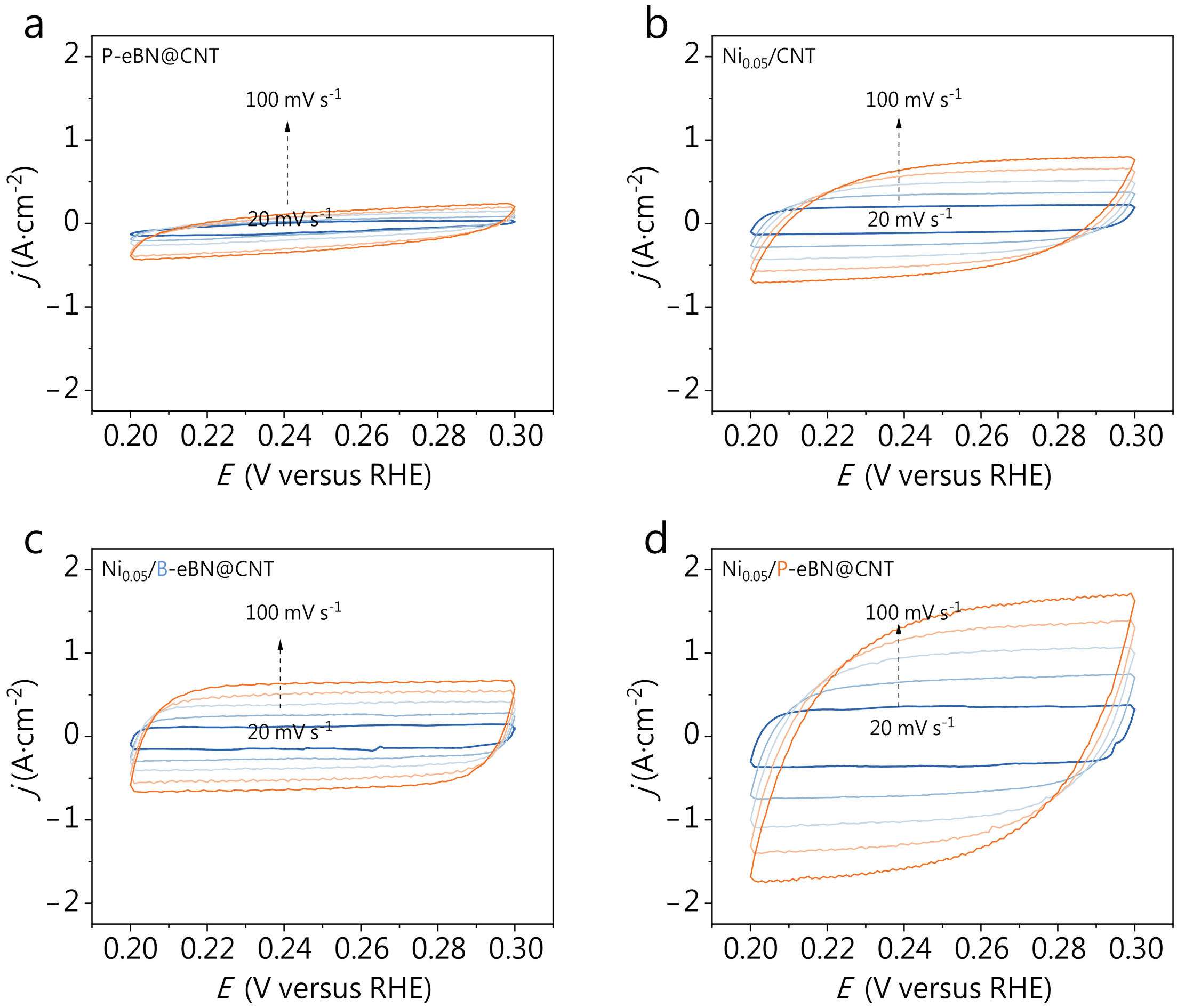


**Fig. S10.** CVs of (a) P-eBN@CNT, (b) $Ni_{0.05}$/CNT, (c) $Ni_{0.05}$/B-eBN@CNT and (d) $Ni_{0.05}$/P-eBN@CNT in 0.5 M $KHCO_3$ solution at different scan rates.

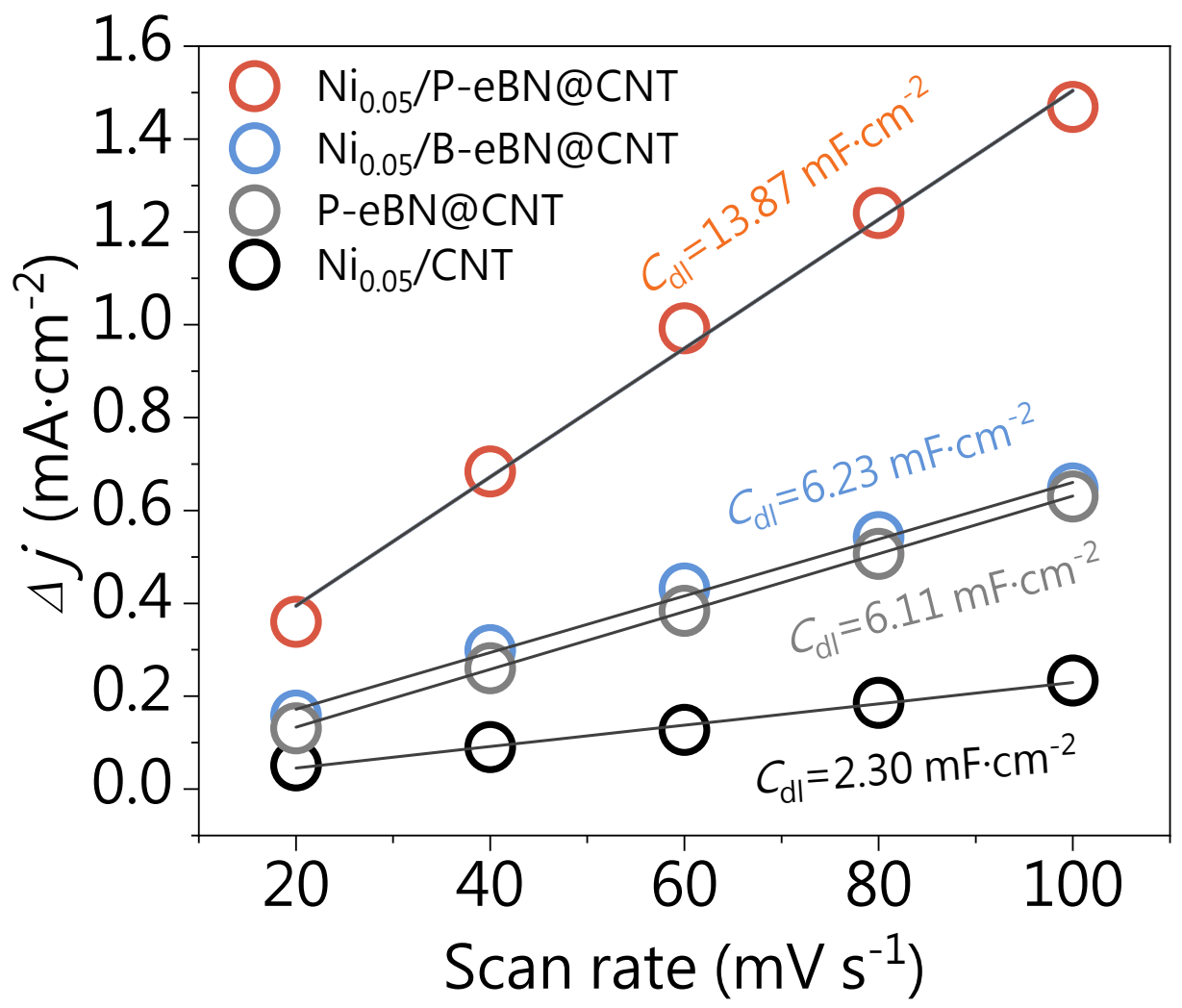


**Fig. S11.** $C_{dl}$ plots of P-eBN@CNT, $Ni_{0.05}$/CNT, $Ni_{0.05}$/B-eBN@CNT and $Ni_{0.05}$/P-eBN@CNT in H-cell.

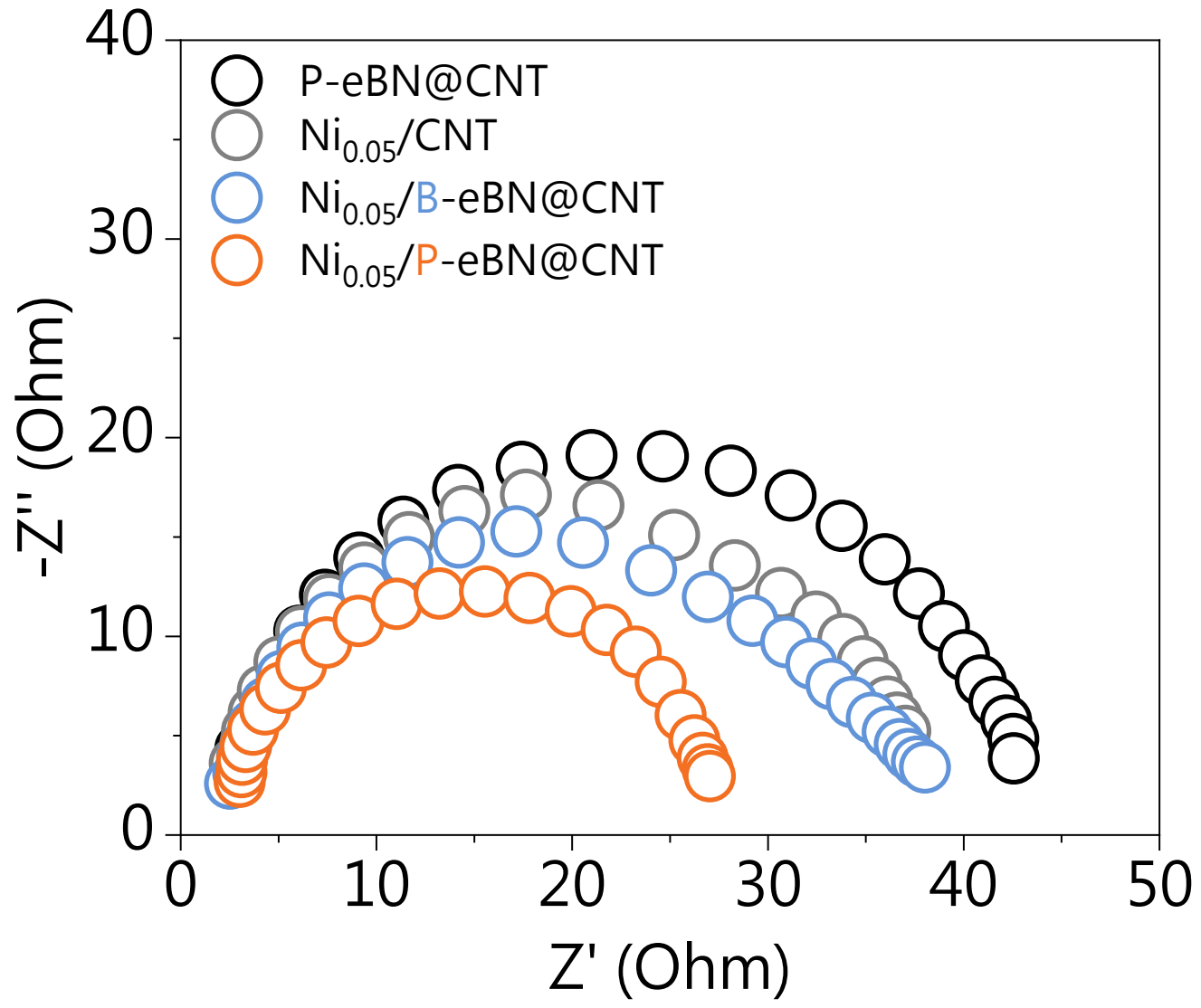


**Fig. S12.** Nyquist plot curves of P-eBN@CNT, $Ni_{0.05}$/CNT, $Ni_{0.05}$/B-eBN@CNT and $Ni_{0.05}$/P-eBN@CNT in H-cell.

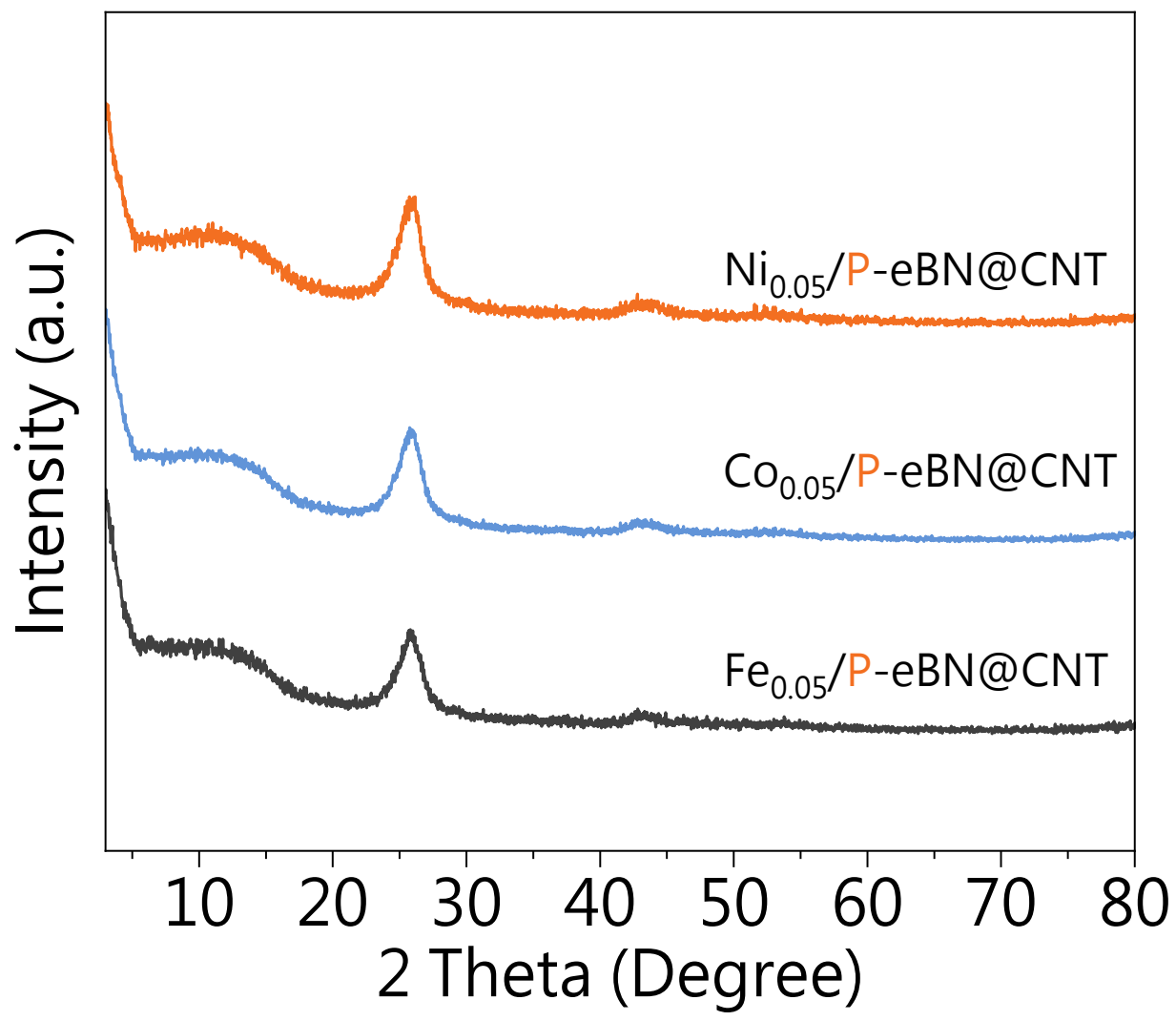


**Fig. S13.** PXRD patterns of $Ni_{0.05}$/P-eBN@CNT, $Fe_{0.05}$/P-eBN@CNT and $Co_{0.05}$/P-eBN@CNT.

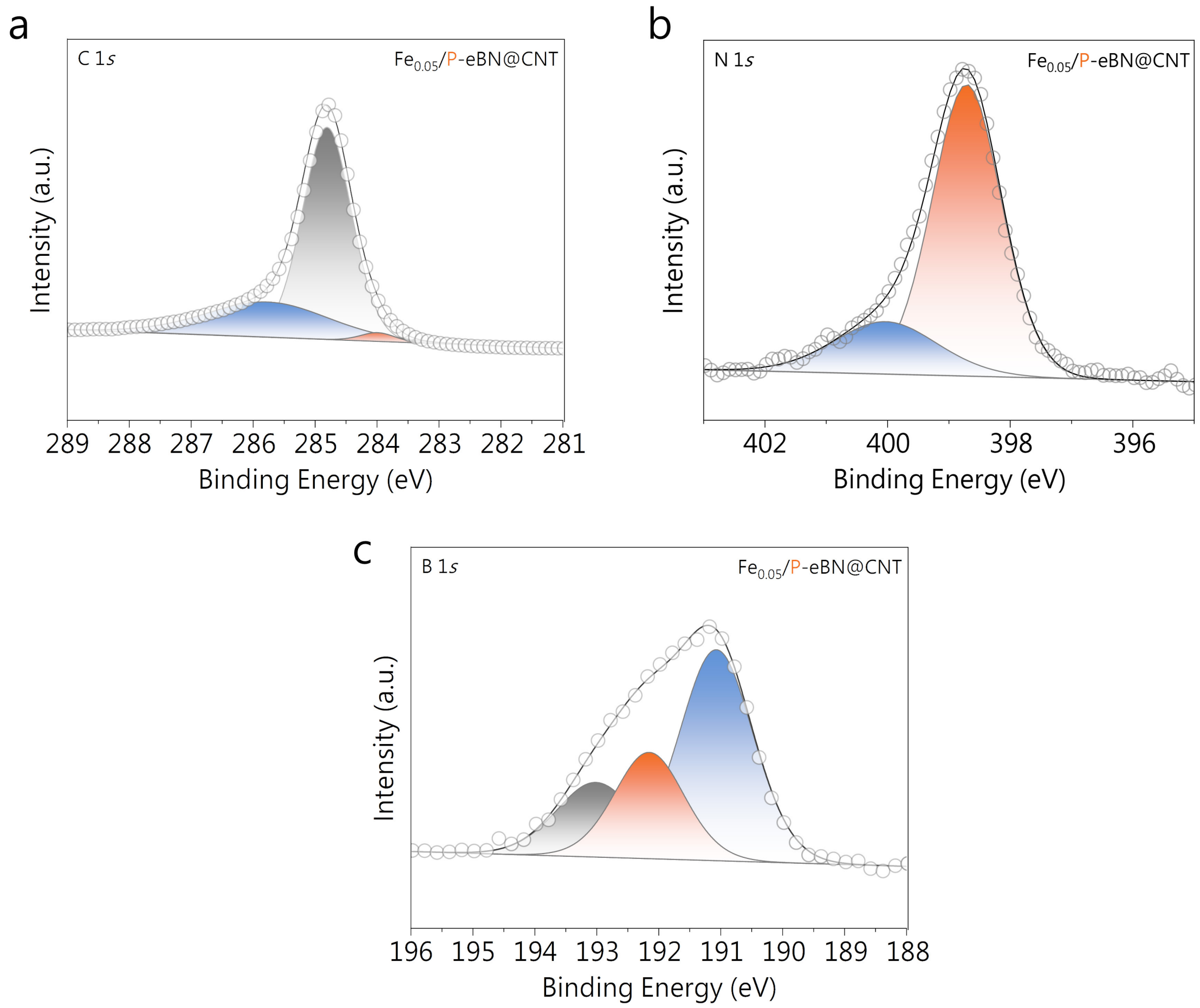


**Fig. S14.** High-resolution XPS spectra of $Fe_{0.05}$/P-eBN@CNT of (a) C 1*s*, (b) N 1*s* and (c) B 1*s*.

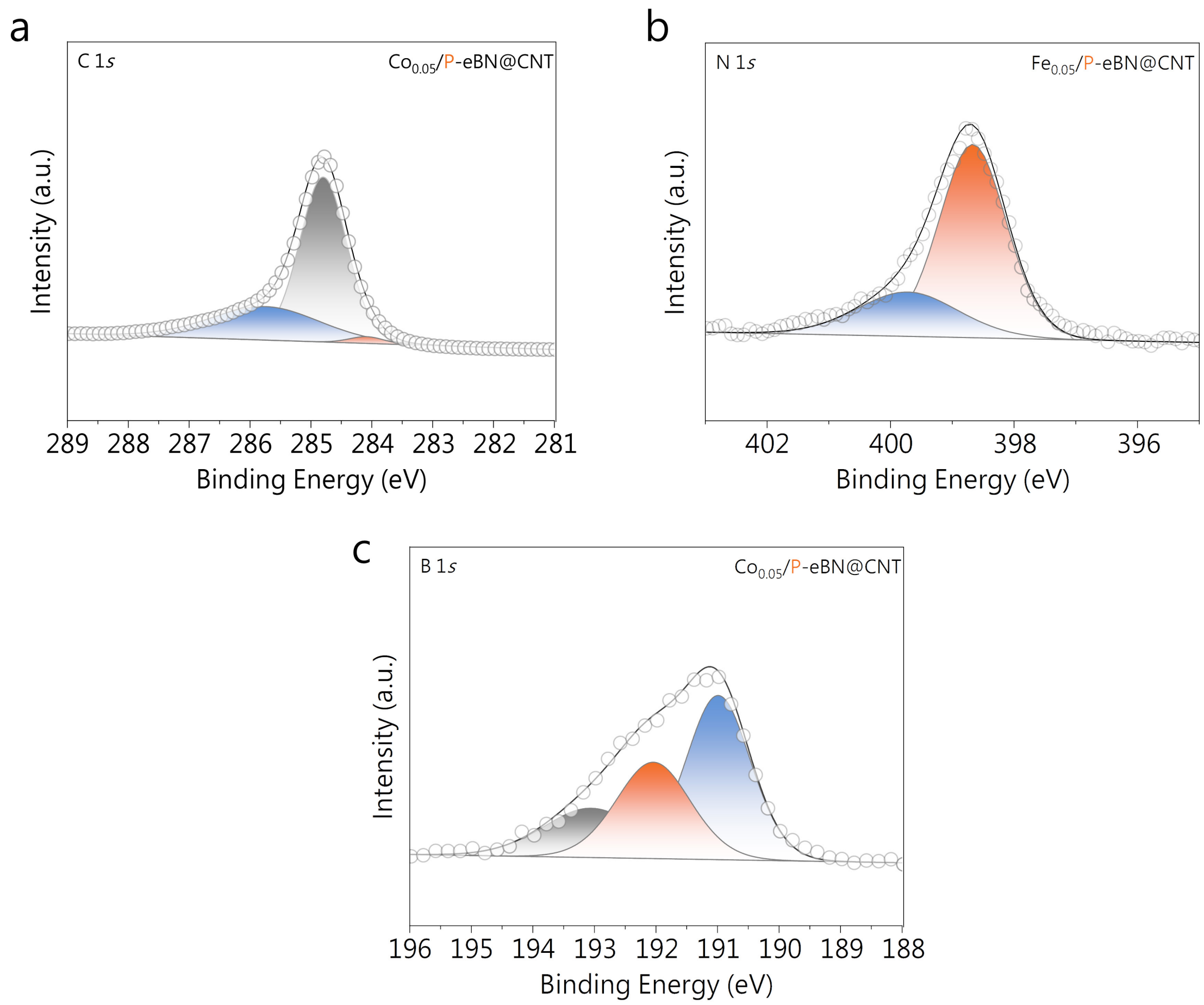


**Fig. S15.** High-resolution XPS spectra of $Co_{0.05}$/P-eBN@CNT of (a) C 1*s*, (b) N 1*s* and (c) B 1*s*.

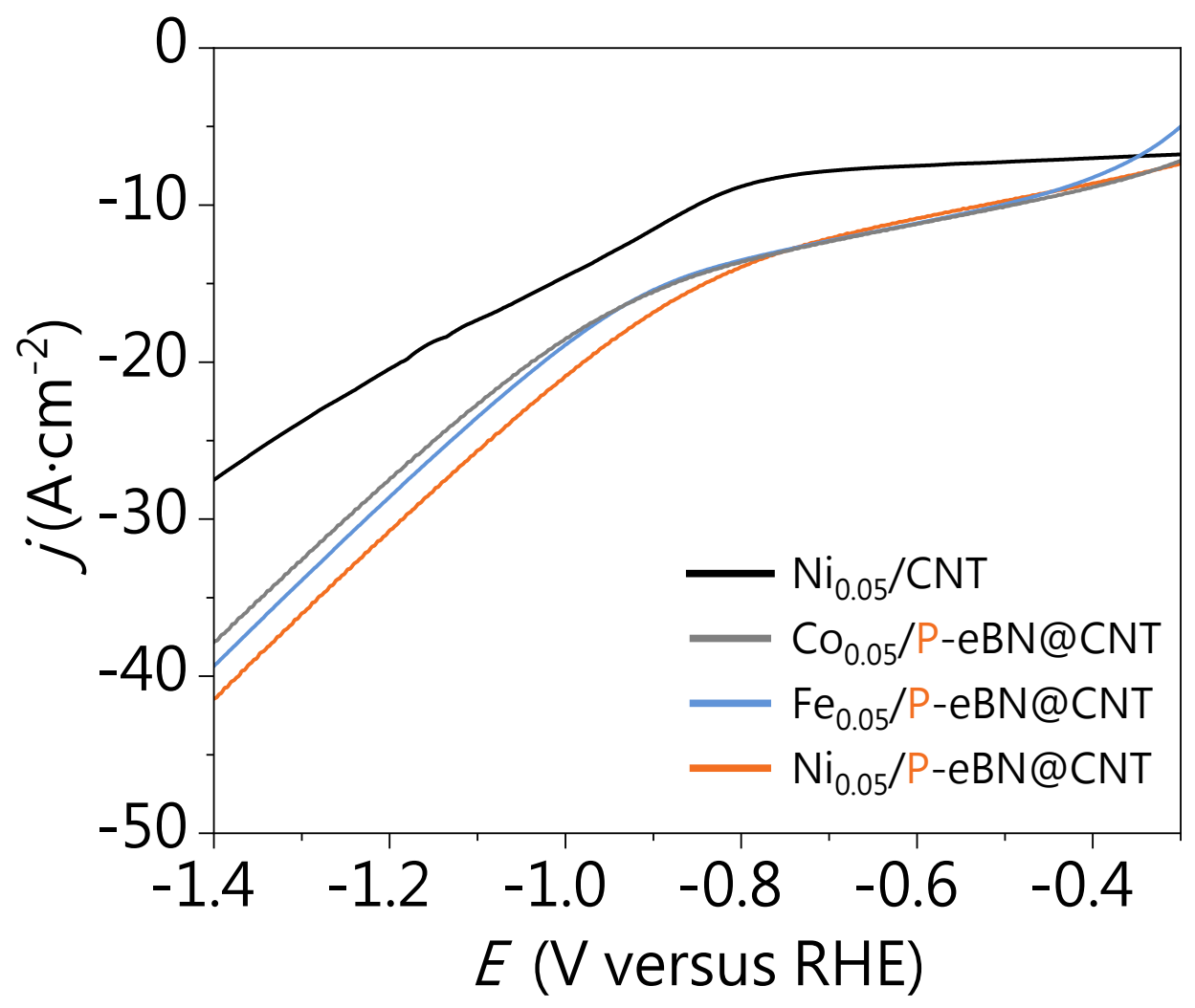


**Fig. S16.** LSV curves of $Ni_{0.05}$/P-eBN@CNT, $Fe_{0.05}$/P-eBN@CNT, $Co_{0.05}$/P-eBN@CNT and $Ni_{0.05}$/CNT tested in H-cell.

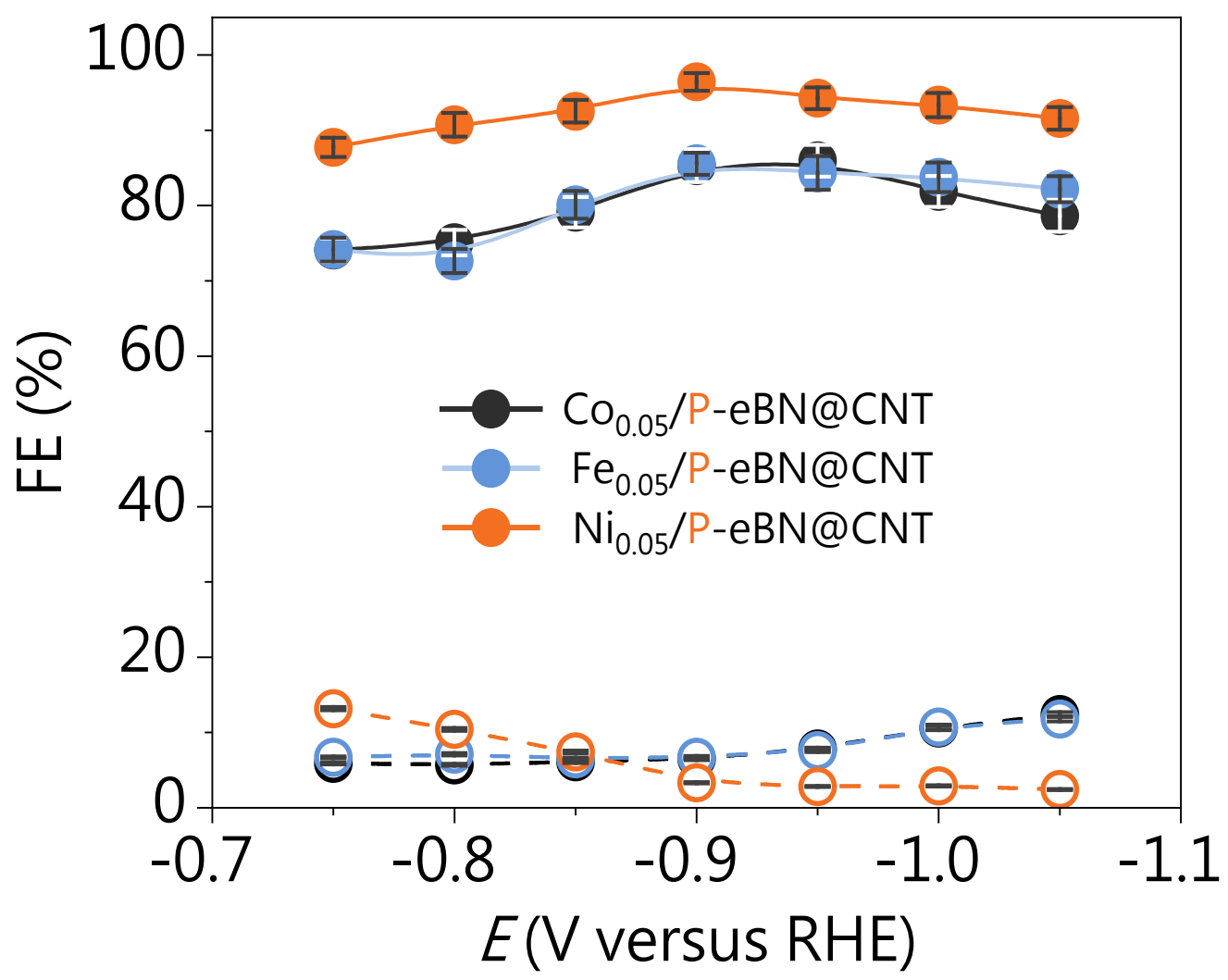


**Fig. S17.** Faradaic efficiencies of $Ni_{0.05}$/P-eBN@CNT, $Fe_{0.05}$/P-eBN@CNT and $Co_{0.05}$/P-eBN@CNT tested in H-cell. Solid lines correspond to CO, while dashed lines stand for $H_2$.

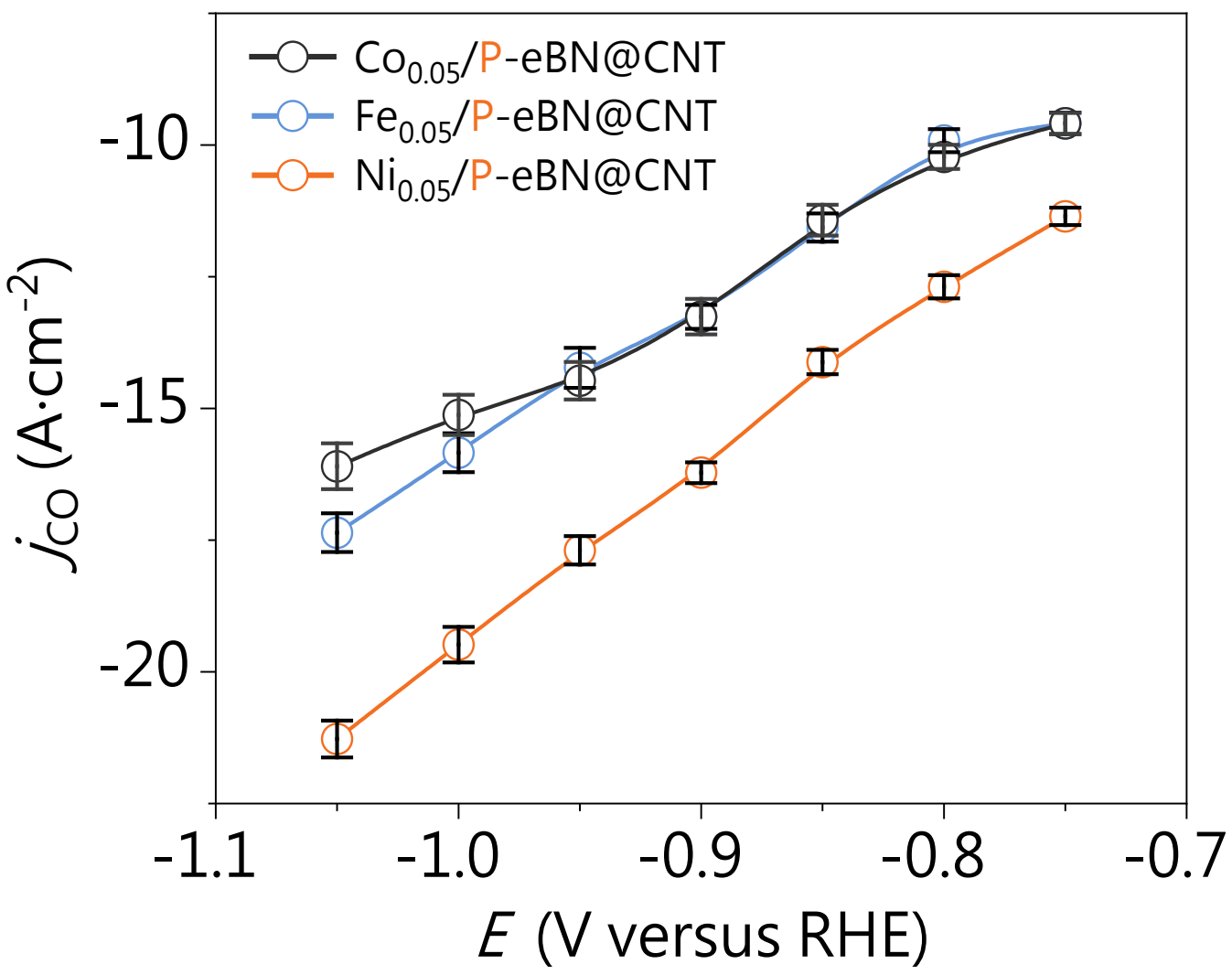


**Fig. S18.** $j_{CO}$ of $Ni_{0.05}$/P-eBN@CNT, $Fe_{0.05}$/P-eBN@CNT and$Co_{0.05}$/P-eBN@CNT tested in H-cell.

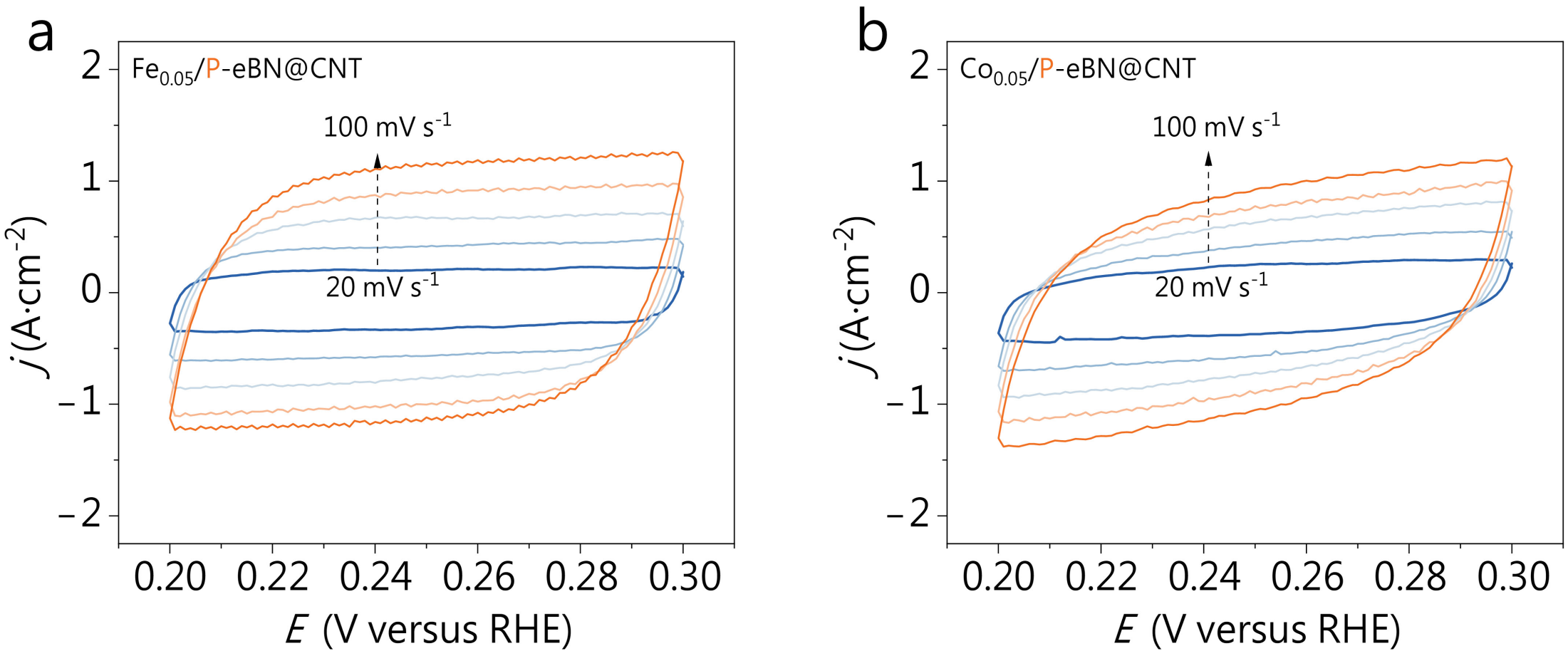


**Fig. S19.** CVs of (a) $Fe_{0.05}$/P-eBN@CNT and (b) $Co_{0.05}$/ CNT in 0.5 M $KHCO_3$ solution at different scan rates.

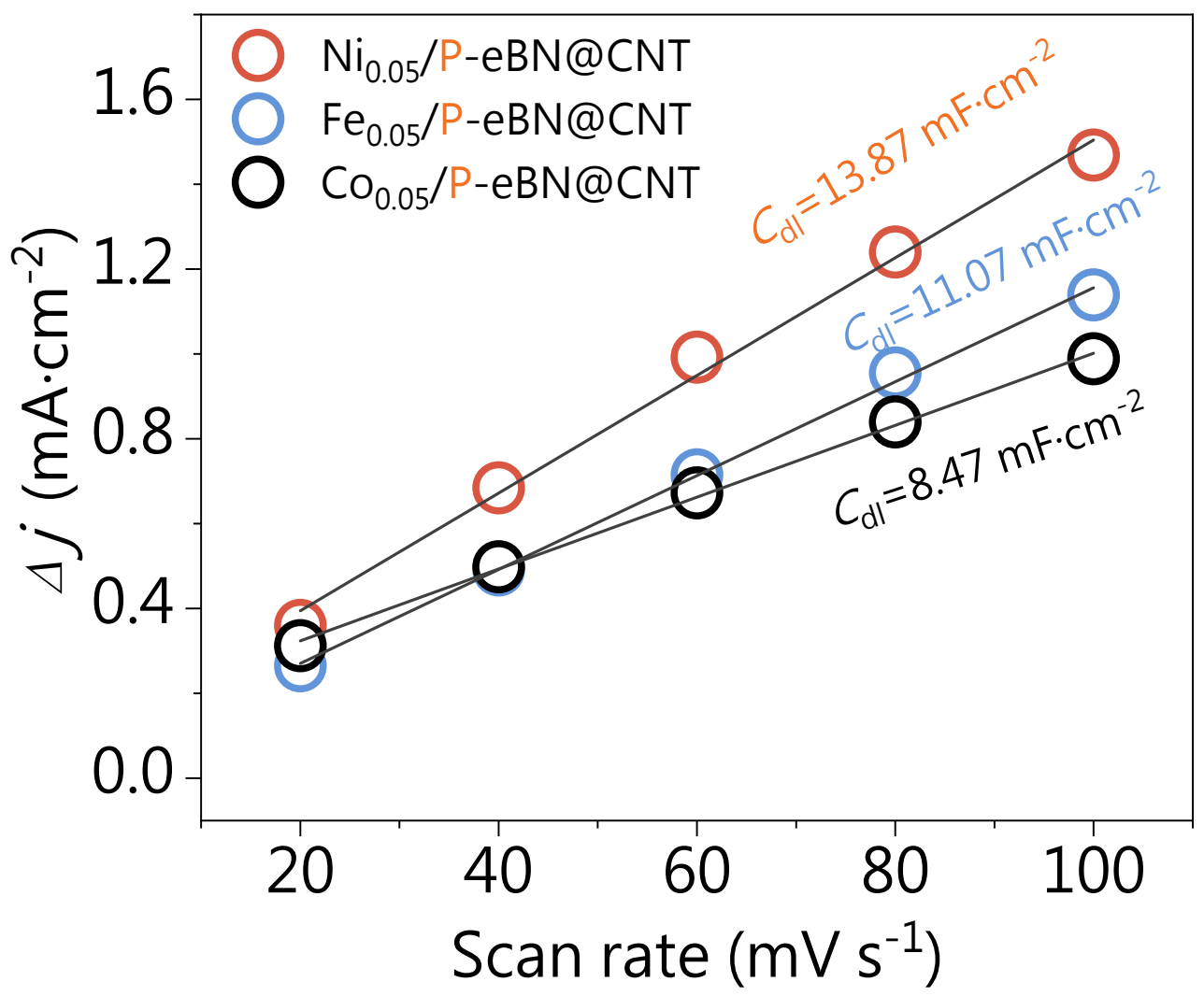


**Fig. S20.** $C_{dl}$ plots of $Ni_{0.05}$/P-eBN@CNT, $Fe_{0.05}$/P-eBN@CNT and $Co_{0.05}$/P-eBN@CNT in H-cell.

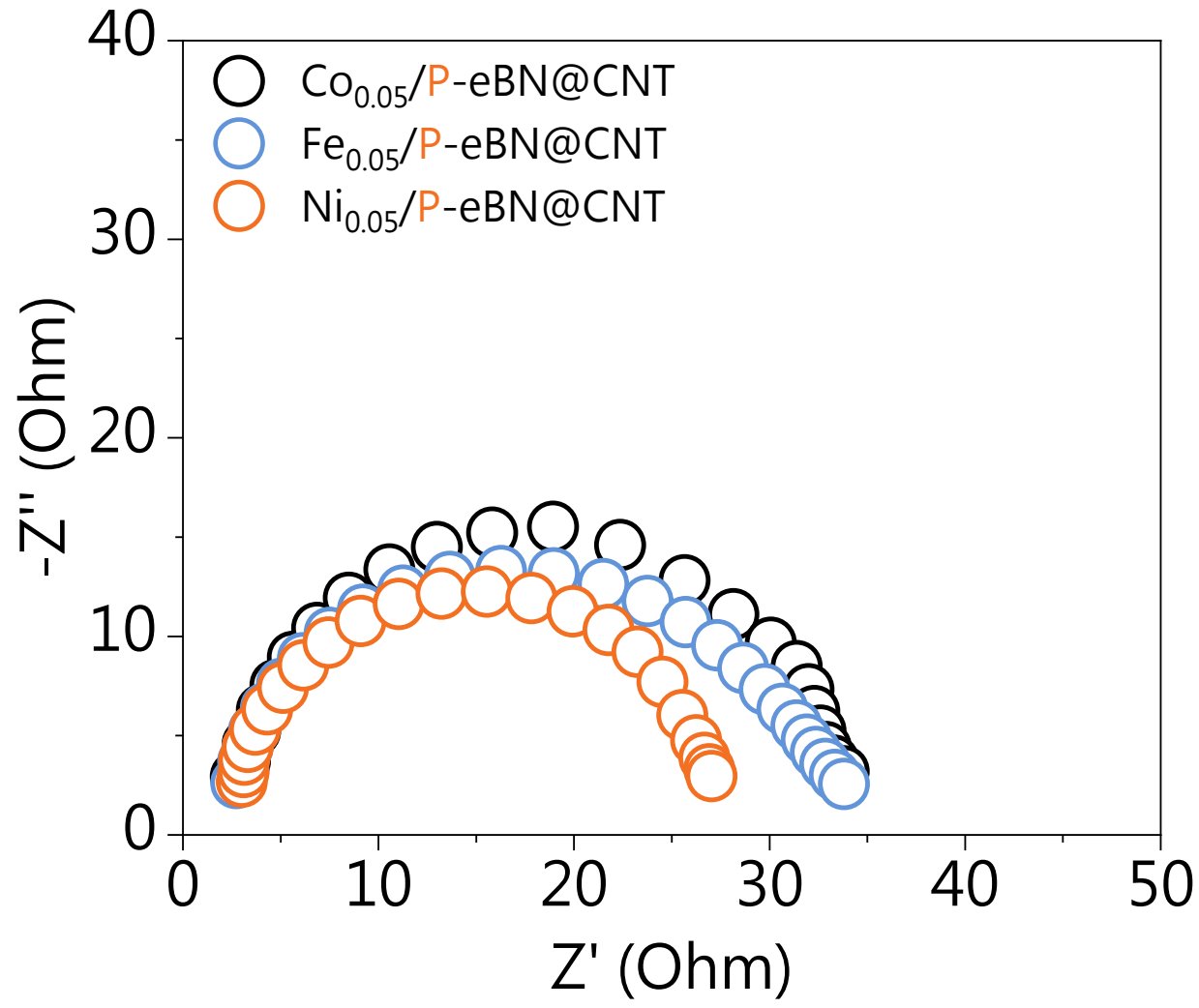


**Fig. S21.** Nyquist plot curves of $Ni_{0.05}$/P-eBN@CNT, $Fe_{0.05}$/P-eBN@CNT and $Co_{0.05}$/P-eBN@CNT in H-cell.

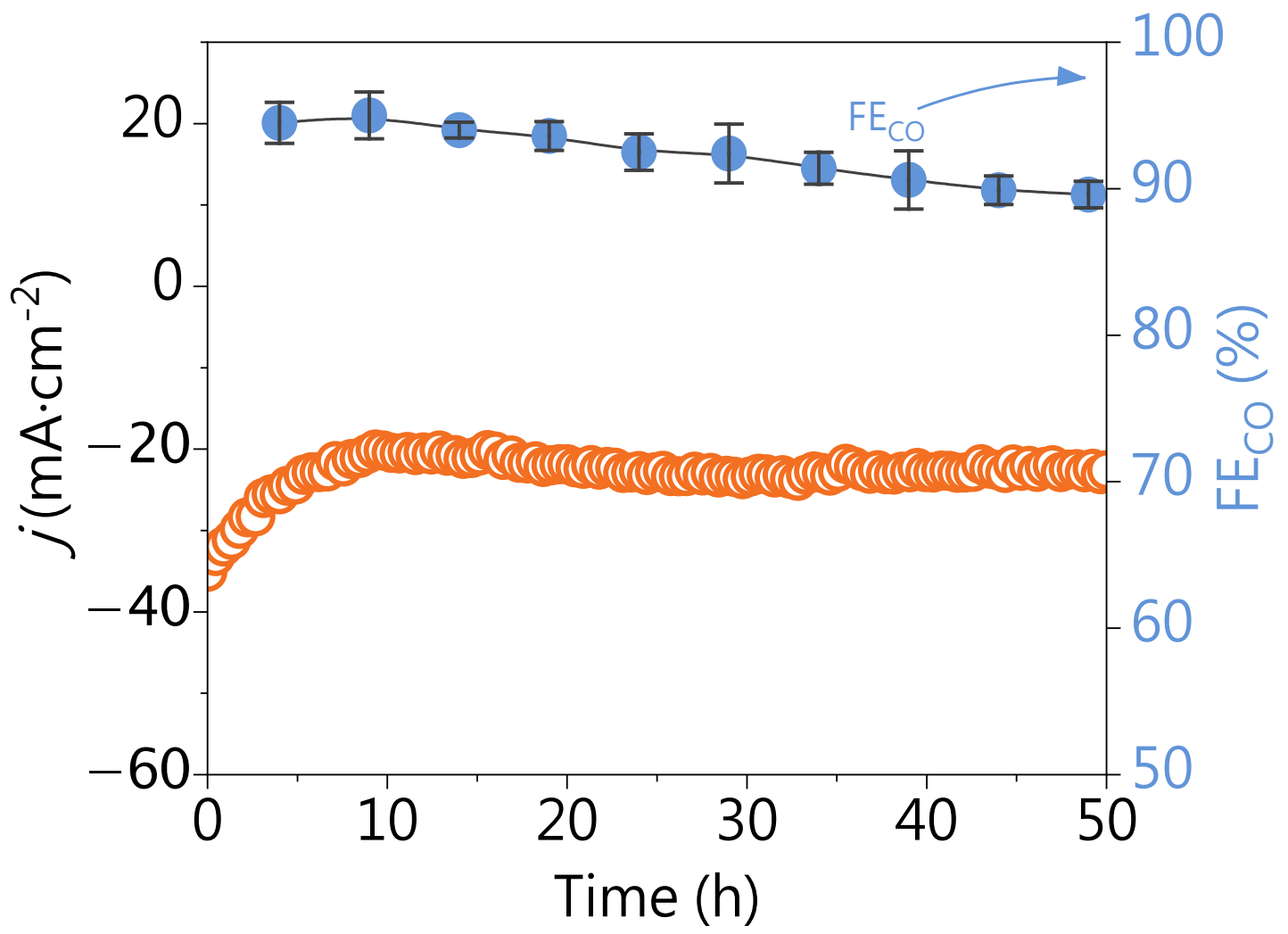


**Fig. S22.** Long-term potentiostatic test and the corresponding formate Faraday efficiencies of $Ni_{0.05}$/P-eBN@CNT at −0.90 V vs. RHE.

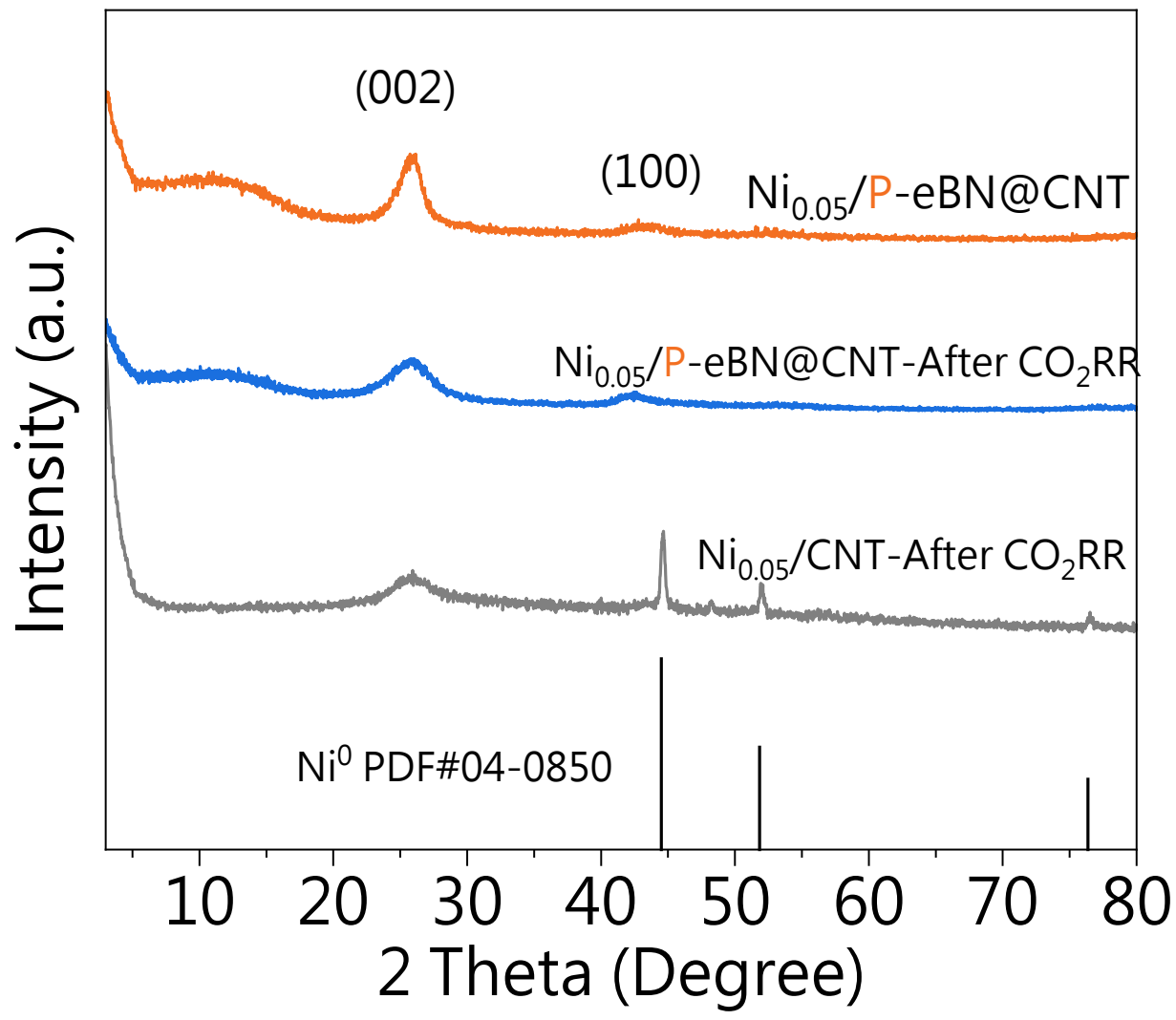


**Fig. S23.** PXRD patterns of $Ni_{0.05}$/P-eBN@CNT, $Ni_{0.05}$/P-eBN@CNT-After $CO_2$RR and $Ni_{0.05}$/CNT-After $CO_2$RR.

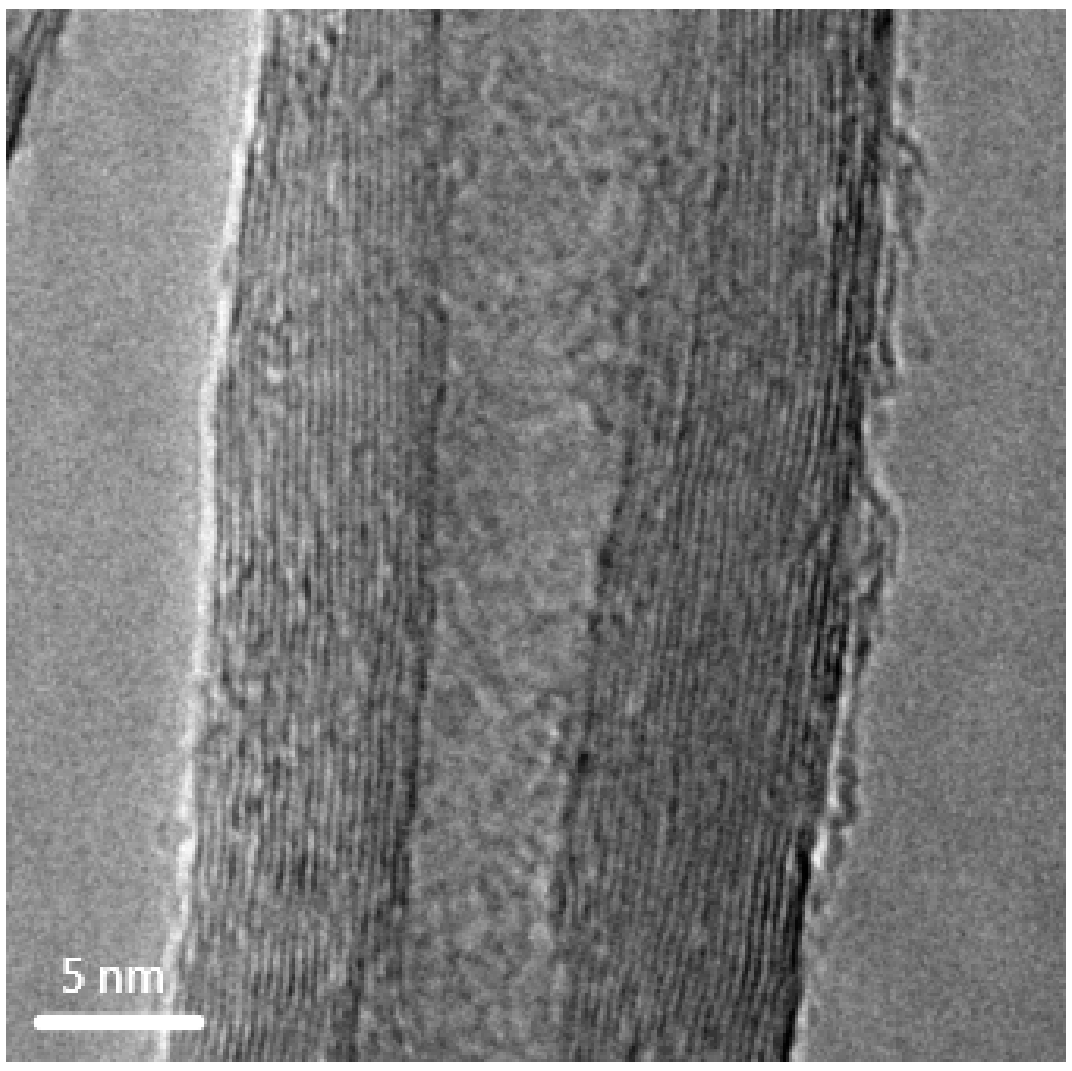


**Fig. S24.** TEM image of $Ni_{0.05}$/P-eBN@CNT-After $CO_2$RR.

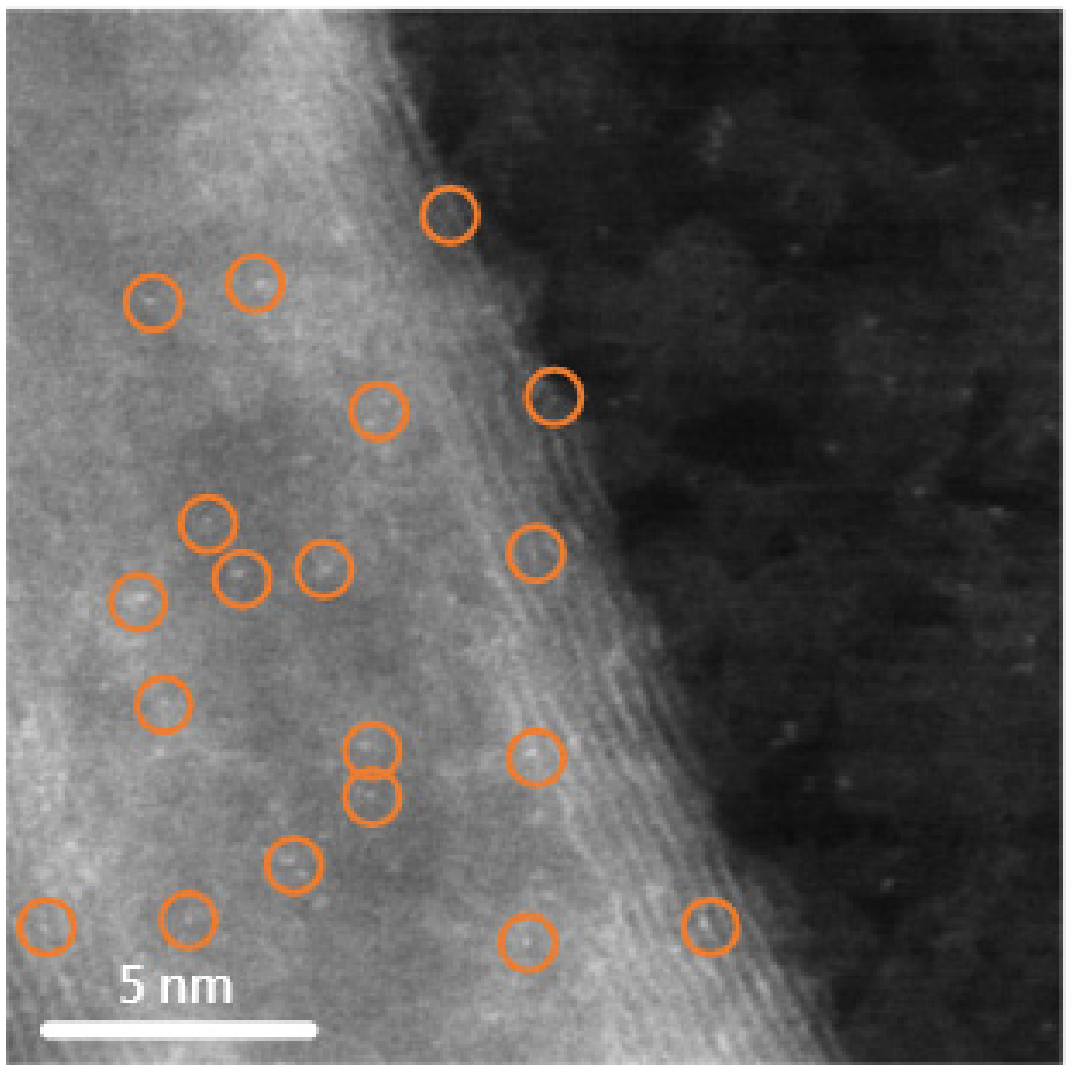


**Fig. S25.** AC-HAADF-STEM images of $Ni_{0.05}$/P-eBN@CNT-After $CO_2$RR.

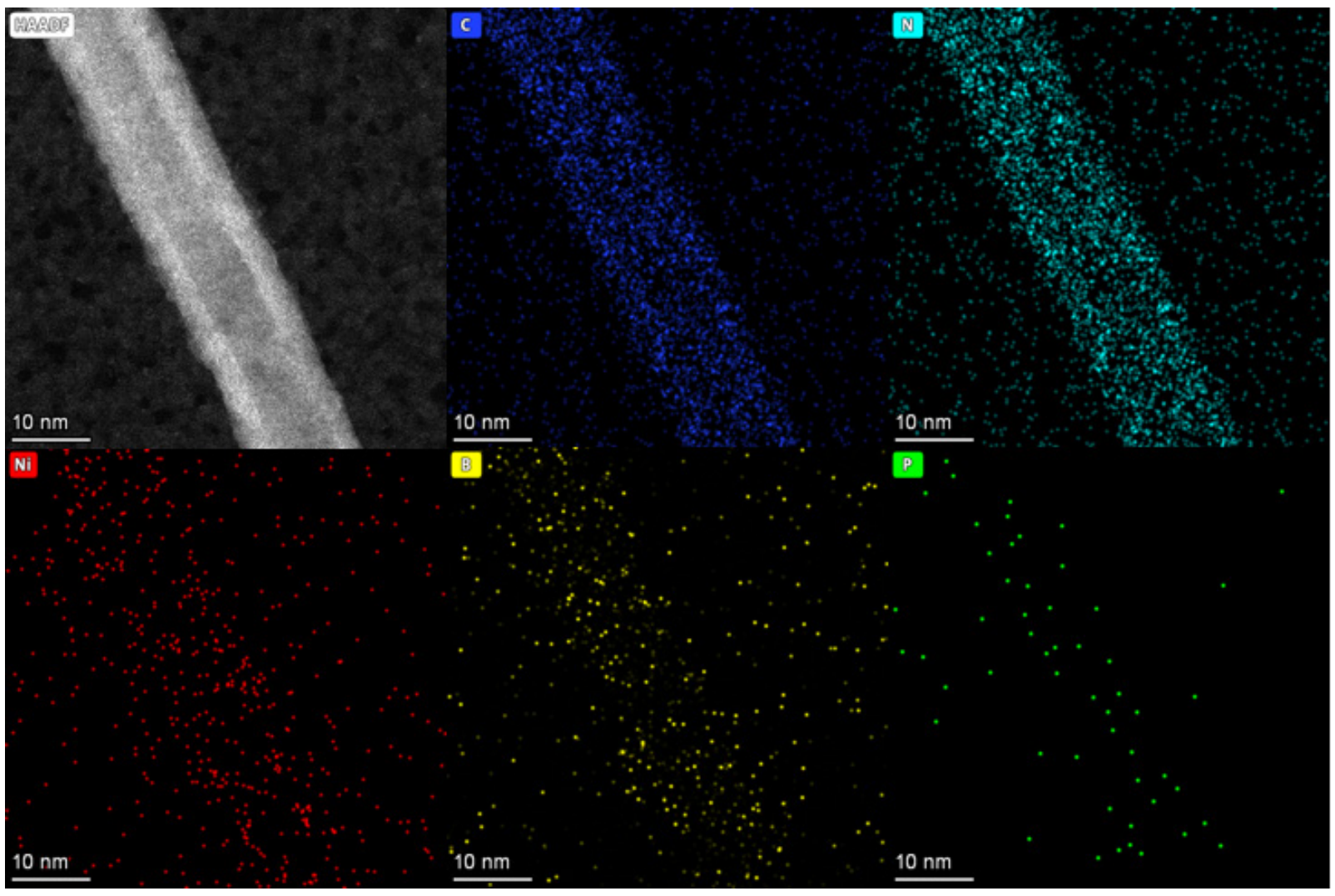


**Fig. S26.** EDX elemental mapping images of $Ni_{0.05}$/P-eBN@CNT-After $CO_2$RR.

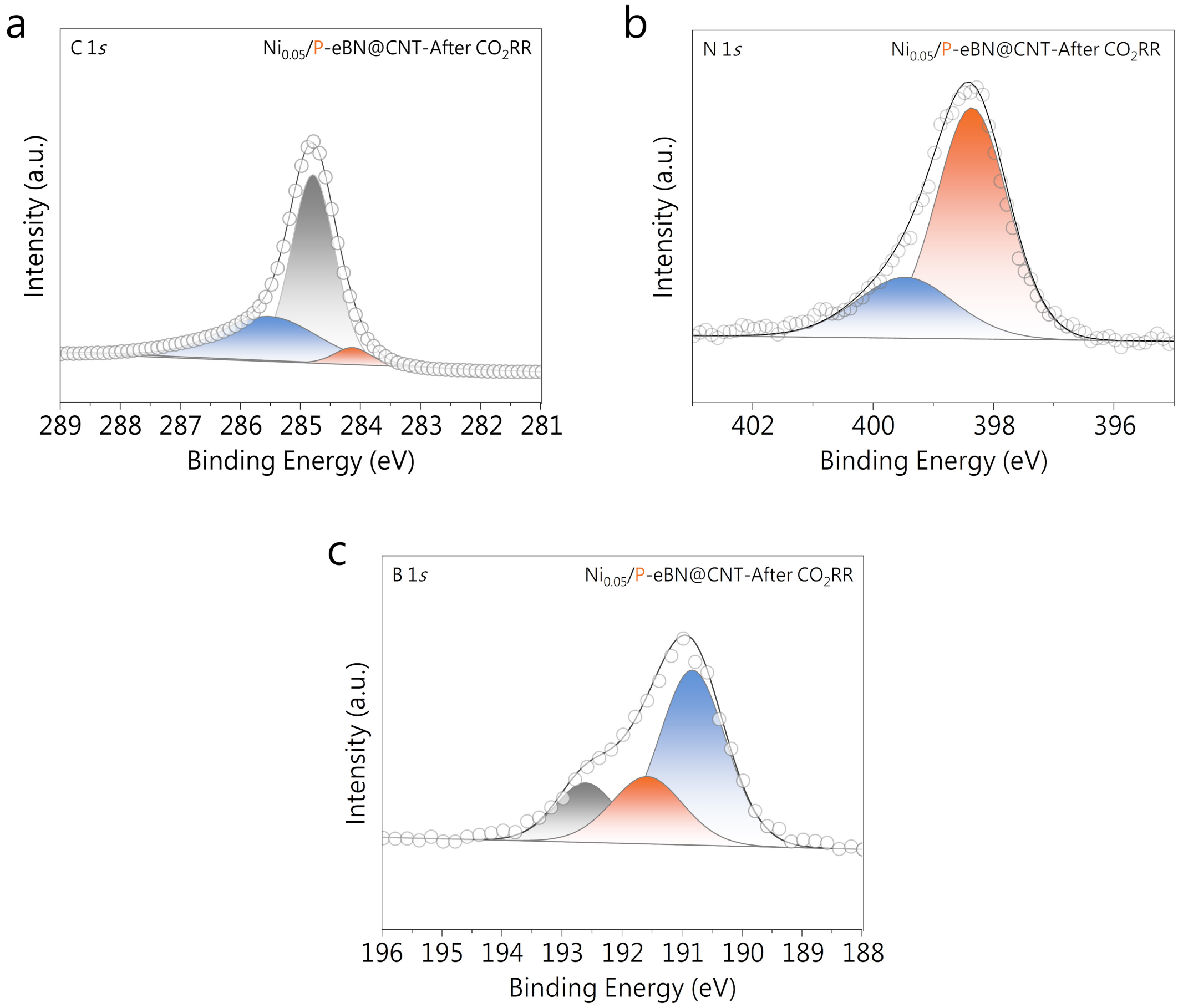


**Fig. S27.** High-resolution XPS spectra of $Ni_{0.05}$/P-eBN@CNT-After $CO_2$RR of (a) C 1*s*, (b) N 1*s* and (c) B 1*s*.

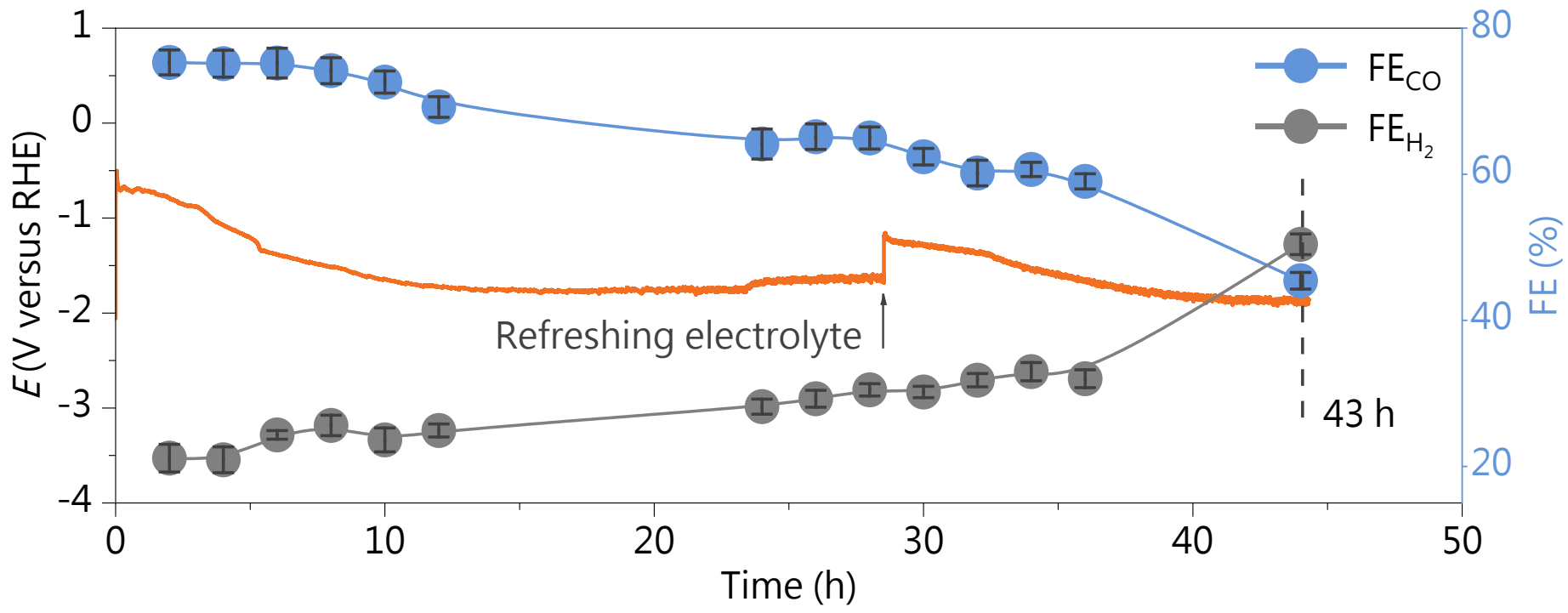


**Fig. S28.** Long-term chronopotentiometry profile together with matched product FE for $Ni_{0.05}$/CNT under 150 mA·cm$^{-2}$ current density.

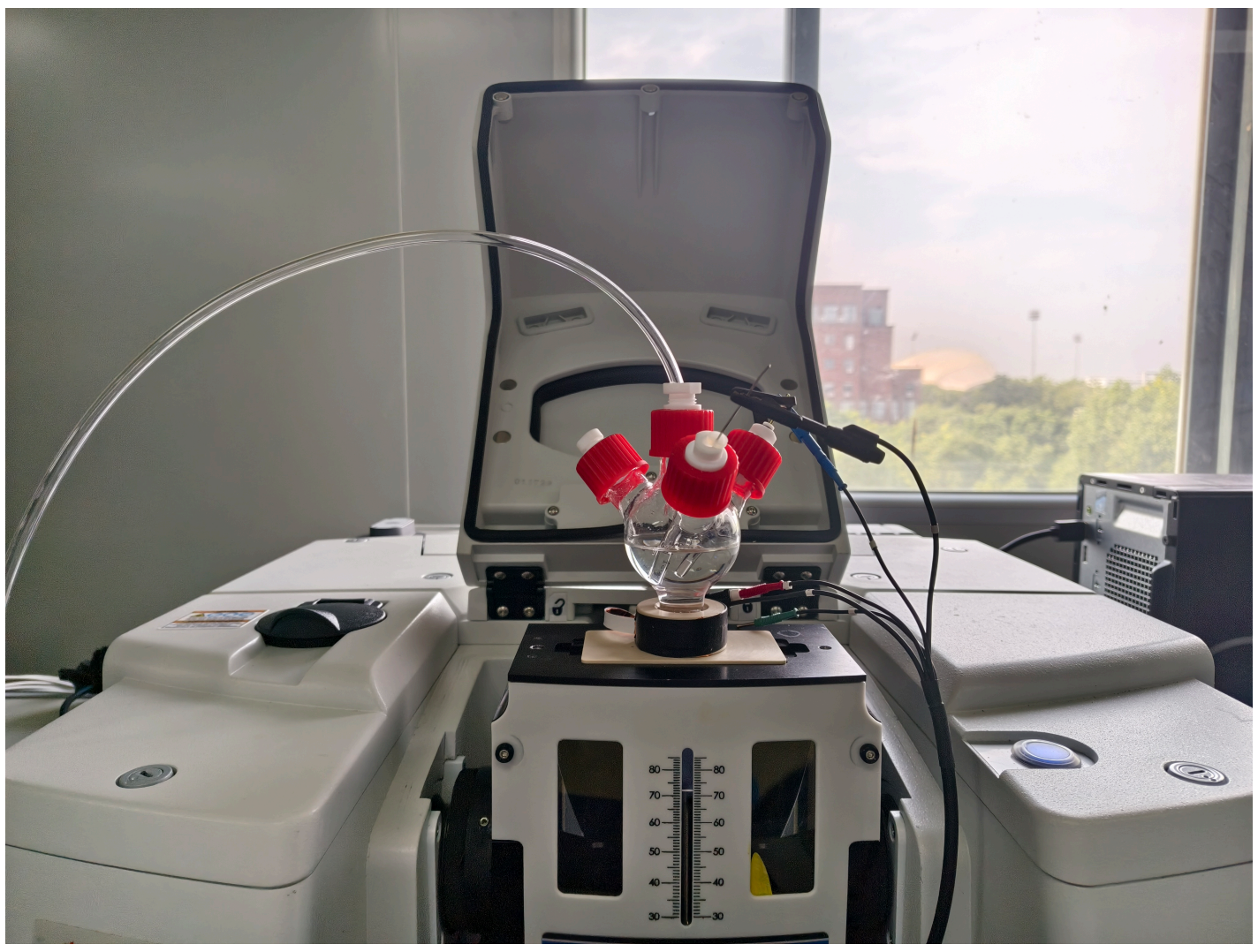

**Fig. S29.** The photograph of the operando ATR-SEIRAS system.

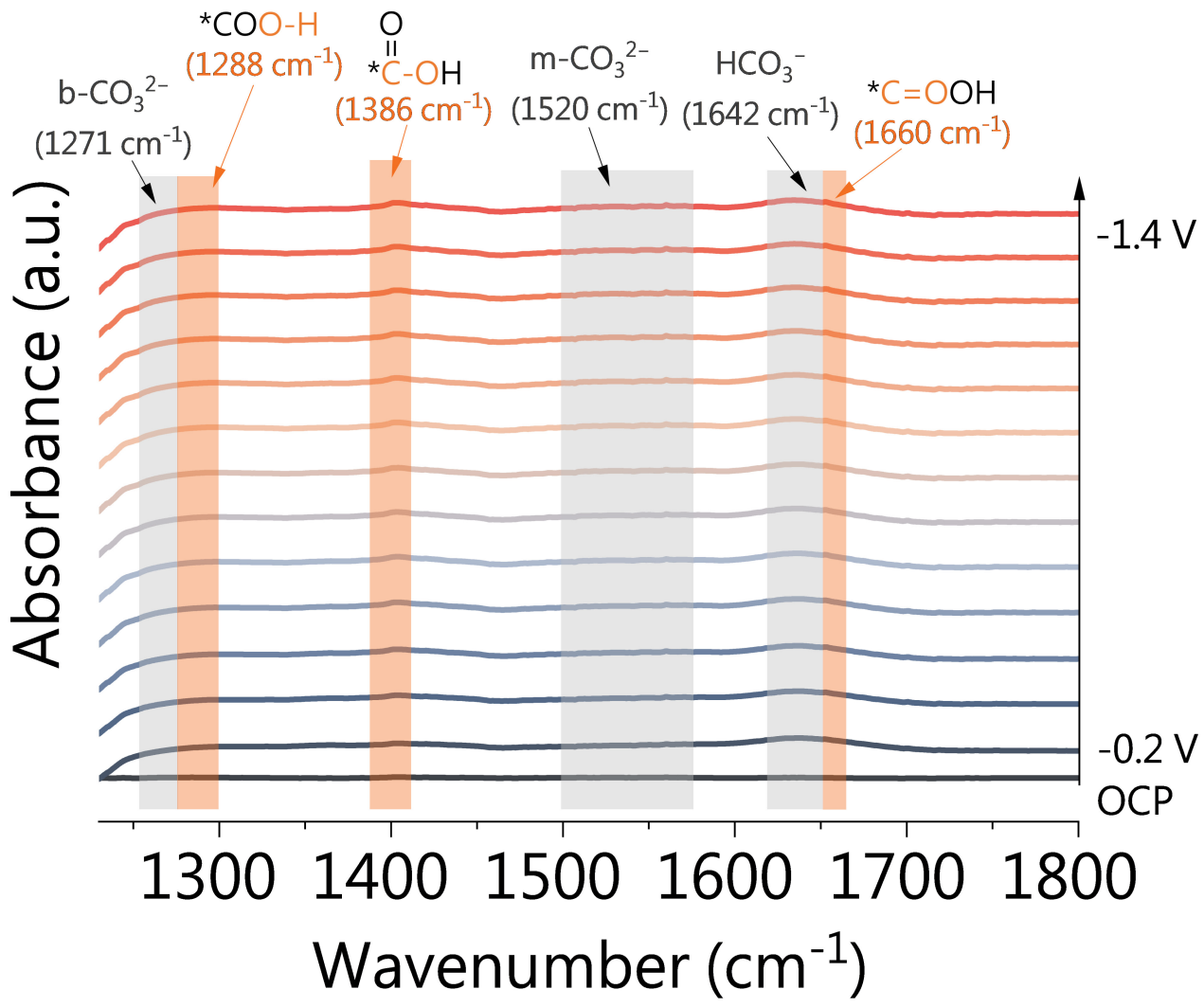


**Fig. S30.** Potential-dependent operando ATR-SEIRAS spectra of $Ni_{0.05}$/B-eBN@CNT from −0.2 to −1.4 V vs. RHE.

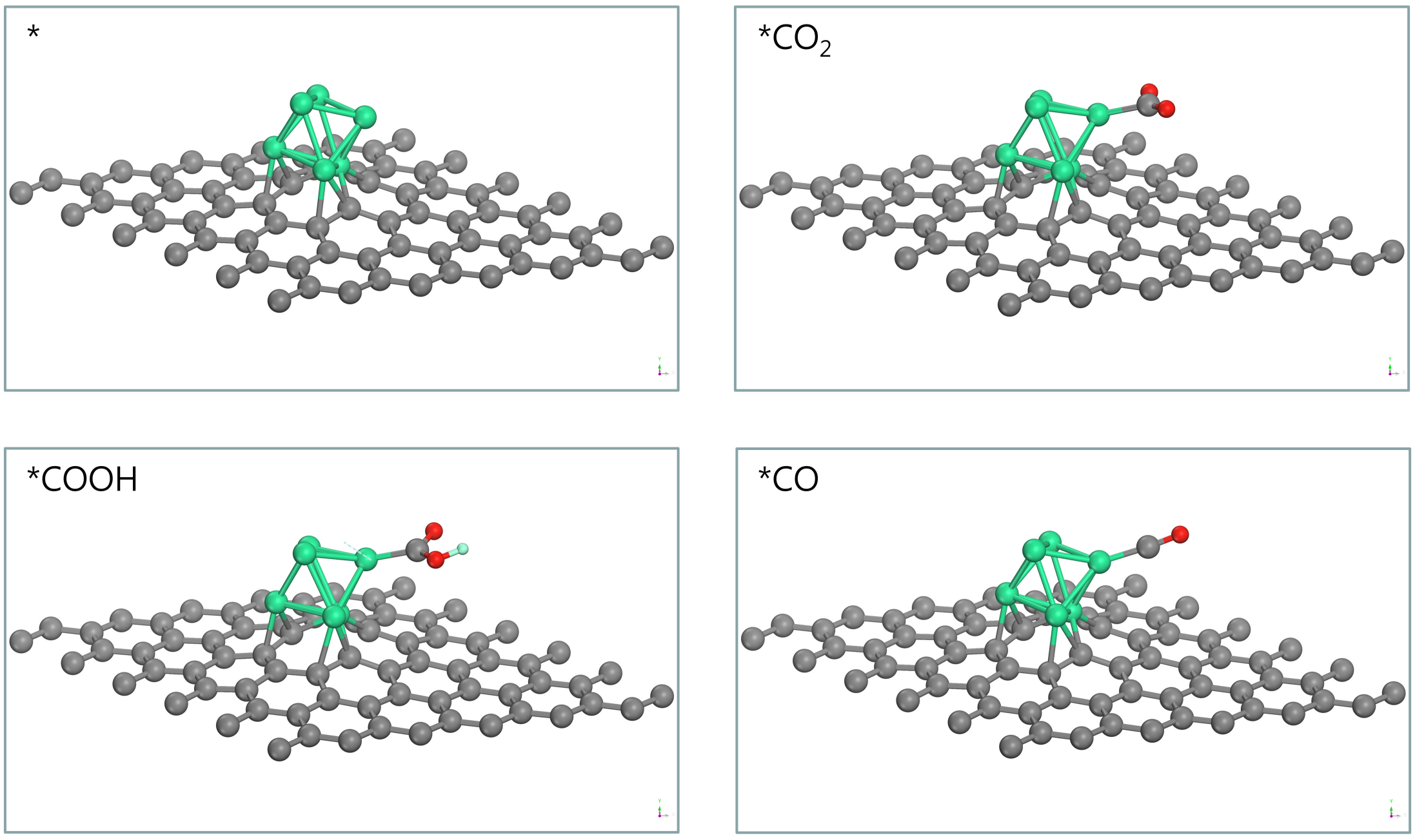


**Fig. S31.** The intermediates and their structural models involved in the electrochemical reduction of $CO_2$ to CO on Ni-NP. Atomic colours: Ni (green), C (black), O (red).

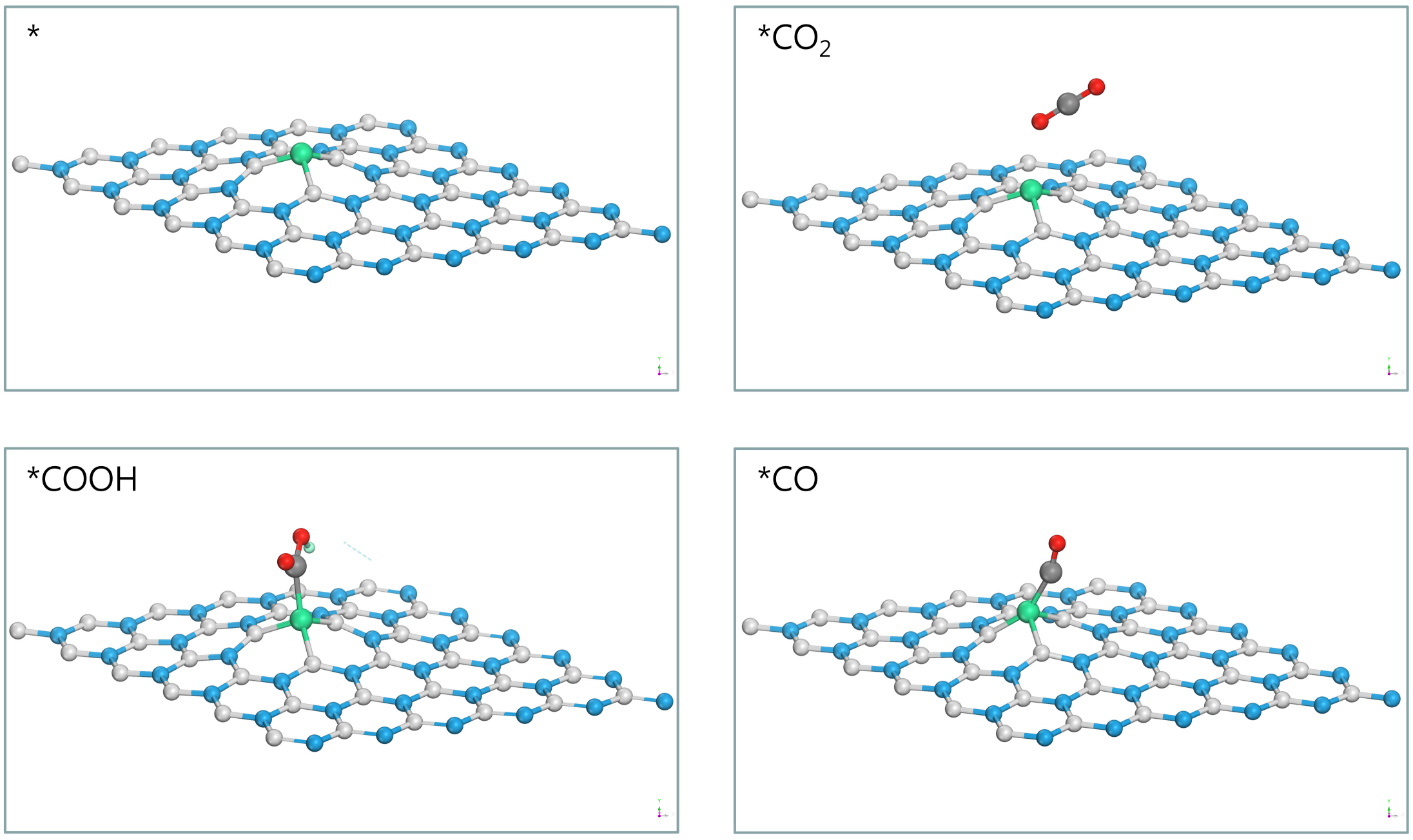


**Fig. S32.** The intermediates and their structural models involved in the electrochemical reduction of $CO_2$ to CO on Ni-SA-eBN. Atomic colours: Ni (green), H (gray), B (blue), C (black), N (white), O (red).

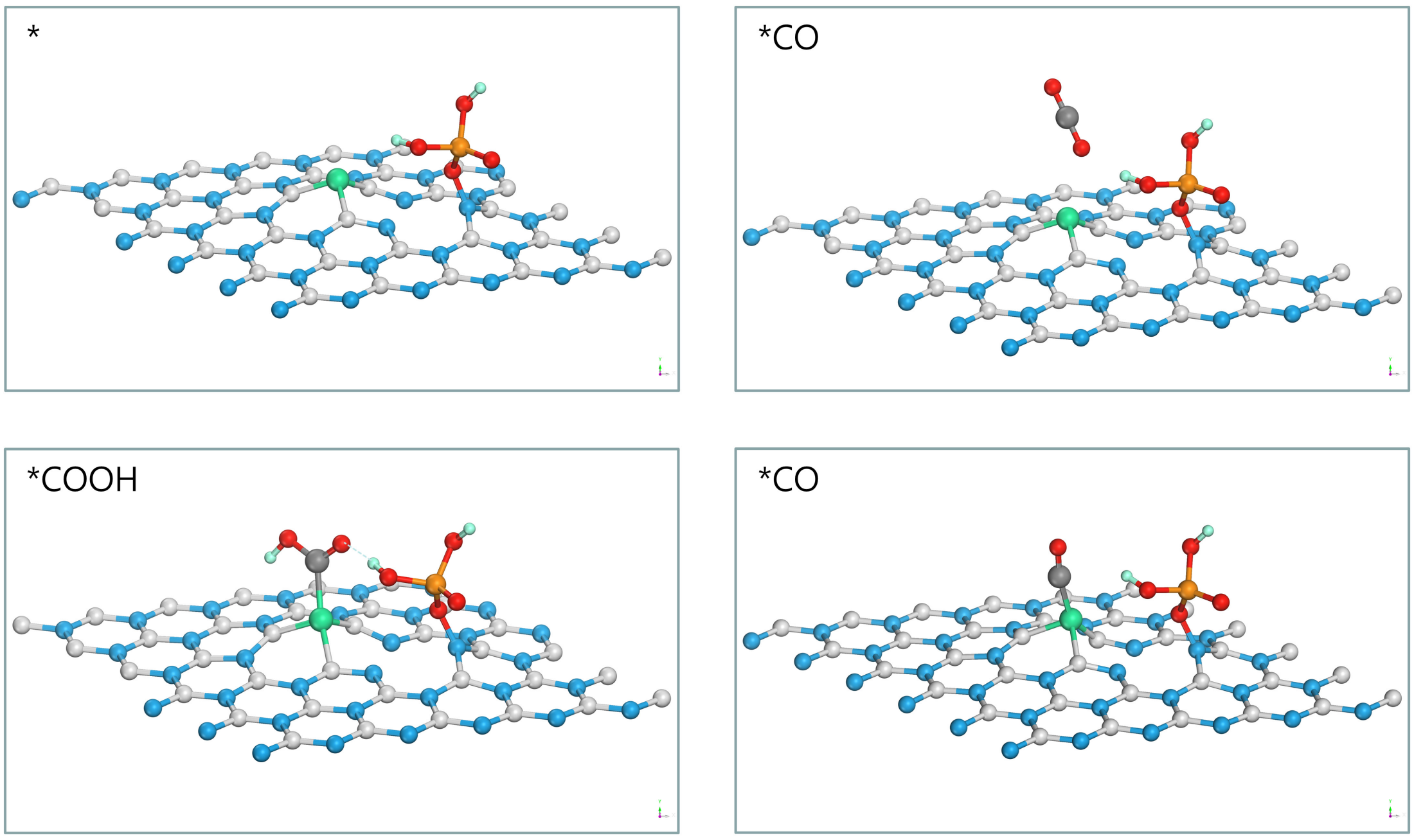


**Fig. S33.** The intermediates and their structural models involved in the electrochemical reduction of $CO_2$ to CO on Ni-SA-P-eBN. Atomic colours: Ni (green), H (gray), B (blue), C (black), N (white), O (red), P (orange).

**Table S1.** ICP-OES data of $Ni_{0.05}$/P-eBN@CNT.

| Material | Ni (wt%) | P (wt%) |
|---|---|---|
| $Ni_{0.05}$/P-eBN@CNT | 0.2965 | 0.1830 |
| $Ni_{0.05}$/B-eBN@CNT | 0.2119 | / |
| $Ni_{0.05}$/CNT | 0.1472 | / |

**Table S2.** EXAFS fitting parameters at the Ni *K*-edge for various samples.

| Sample | Shell | $CN^a$ | $R$(Å)$^b$ | $\sigma^2$(Å$^2$)$^c$ | $\Delta E_0$(eV)$^d$ | $R$ factor |
|---|---|---|---|---|---|---|
| Ni foil | Ni-Ni | 12* | 2.48±0.00 | 0.006 | 6.40±0.88 | 0.003 |
| $Ni_{0.05}$/P-eBN@CNT | Ni-O/N | 2.74±0.22 | 2.04±0.01 | 0.008 | −1.40±0.86 | 0.002 |
| $Ni_{0.05}$/B-eBN@CNT | Ni-O/N | 2.73±0.15 | 2.02±0.01 | 0.006 | −6.98±0.74 | 0.001 |

**Table S3.** Relative Gibbs free energies (eV) of $CO_2$RR intermediates over Ni-SA-P-eBN, Ni-SA-BN and Ni-NP. Relative energy vs. prior step is the energy difference from the preceding intermediate, and relative energy vs. initial species refers to the initial state.

| Structural Model | Step | Relative energy vs. prior step (eV) | Relative energy vs. initial species (eV) |
|---|---|---|---|
| Ni-NP | * + $CO_2$ | / | 0.00 |
| | *$CO_2$ | 0.52 | 0.5 |
| | *COOH | 0.58 | 1.10 |
| | *CO | −0.79 | 0.31 |
| | * + CO | 0.32 | 0.63 |
| Ni-SA-eBN | * + $CO_2$ | / | 0.00 |
| | *$CO_2$ | 0.37 | 0.37 |
| | *COOH | 0.33 | 0.70 |
| | *CO | −0.39 | 0.31 |
| | * + CO | 0.33 | 0.63 |
| Ni-SA-P-eBN | * + $CO_2$ | / | 0.00 |
| | *$CO_2$ | −0.16 | −0.16 |
| | *COOH | 0.39 | 0.22 |
| | *CO | −1.14 | −0.92 |
| | * + CO | 1.56 | 0.63 |

**Table S4.** Comparison of the $CO_2$RR performance of Ni-based catalysts.

| Catalysts | Electrolytes | $FE_{CO}$ (%) | *jco* (mA·cm$^{-2}$) | stability (h) | References |
|---|---|---|---|---|---|
| **$Ni_{0.05}$/P-eBN@CNT** | **0.5 M $KHCO_3$** | **97.6±1.2** | **148.6 ± 2.3** | **87** | **This work** |
| 0.05Ni-DCD-C-1050 | 0.5 M $KHCO_3$ | ~100 | 130 | 10 | [7] |
| Ar-Ni/NC | 0.1 M $KHCO_3$ | ≈90% | 60 | 24 | [8] |
| NiCd-HDAC | 0.1 M $KHCO_3$ | 97.1 ± 0.2% | ~15 | 55 | [9] |
| Ni-N-C | 0.5 M $KHCO_3$ | ≈95% | 56.3 | 50 | [10] |
| $Ni_{1.0\%}$−N−C | 0.1 M $KHCO_3$ | 97 | 12 | 0.5 | [11] |
| Ni@SNC-10 | 0.1 M $KHCO_3$ | 94.6 | 18.3 | 16 | [12] |
| Bi,Ni/N-C | 0.1 M $KHCO_3$ | ≈95% | ~29 | 20 | [13] |
| Ni-N-CF | 0.5 M $NaHCO_3$ | 92 | 2.6 | 12 | [14] |
| 2Ni@N-C-800 | 0.5 M $KHCO_3$ | 93 | 93 | 24 | [15] |
| Ni-SNC | 0.5 M $KHCO_3$ | 95 | 11 | 24 | [16] |
| ACP/S-N-Ni | 0.5 M $KHCO_3$ | 91 | 3.4 | 14 | [17] |
| Pyrrolic N-stabilized Ni SAC | 0.5 M $KHCO_3$ | 98.2 | 148.25 | 24 | [18] |
| ZnNi-DCN-1000 | 0.5 M $KHCO_3$ | 95 | ~143 | 20 | [19] |
| Ni-NG-900 | 0.5 M $KHCO_3$ | 96.5 | 59.6 | 24 | [20] |
| Ni–Zn–N–C | 0.5 M $KHCO_3$ | 99 | 20.23 | 28 | [21] |
| Ni-N/PC-900 | 1 M KOH | 98 | 245 | 8 | [22] |
| NiFe-N-C/BMImP$F_6$ | 0.5 M $KHCO_3$ | 91.9 | 120 | 10 | [23] |
| Ni@GNRs | 0.5 M $KHCO_3$ | 96 | 44 | 20 | [24] |

| | | | | | |
|---|---|---|---|---|---|
| I-Ni SA/NHCRs | 0.5 M $NaHCO_3$ | 94.91 | 15.35 | 13 | [25] |
| Mn─Ni─NC | 0.5 M $KHCO_3$ | 98.7 | 16.8 | 24 | [26] |
| Ni@NiNCM | 0.5 M $KHCO_3$ | 97.6 | 100 | 10 | [27] |
| Ni-N-C-900 | 0.5 M $KHCO_3$ | 92 | 92.1 | 70 | [28] |

## References


1 G. Kresse and J. Hafner, *Phys. Rev. B*, 1993, **47**, 558–561.

2 G. Kresse, *Phys. Rev. B*, 1996, **54**, 11169–11186.

3 P. E. Blöchl, *Phys. Rev. B*, 1994, **50**, 17953–17979.

4 J. P. Perdew, K. Burke and M. Ernzerhof, *Phys. Rev. Lett.*, 1996, **77**, 3865–3868.

5 H. J. Monkhorst and J. D. Pack, *Phys. Rev. B*, 1976, **13**, 5188–5192.

6 V. Wang, N. Xu, J.-C. Liu, G. Tang and W.-T. Geng, *Comput. Phys. Commun.*, 2021, **267**, 108033.

7 P. Pang, C. Xu, R. Li, N. Gao, X. Du, T. Li, J. Wang and G. Xiao, *J. Fuel Chem. Technol.*, 2025, **53**, 1162–1172.

8 X. Li, T. Gan, X. Gao, B. Li, J. Peng, Y. Ji, S. Chen, J. Zhang, J. Zhang, P. K. Das, V. Ramalingam, M. Zhang, P. Zhang, K. Peramaiah and Y. Qiu, *Small*, 2025, **21**, e05521.

9 Z. Yao, H. Cheng, Y. Xu, X. Zhan, S. Hong, X. Tan, T.-S. Wu, P. Xiong, Y.-L. Soo, M. M.-J. Li, L. Hao, L. Xu, A. W. Robertson, B. Xu, M. Yang and Z. Sun, *Nat. Commun.*, 2024, **15**, 9981.

10 Y. Li, J. Zhang, Z. Cao, S. Liu, J. Zhao, M. Lyons, J. Yang, Z. Feng, M. Qiu, Y. C. Li and G. Wu, *Appl. Catal., B: Environ.*, 2026, **399**, 127083.

11 J. C. Weiss, Y. He, D. A. Cullen, A. Benavidez, J. D. Jernigen, H. Zhang, L. Osmieri and P. Zelenay, *Small*, 2025, **21**, 2412162.

12 Y. Zhang, Y. Feng, M. Zhang, Z. Yu, X. Sun and L. Zhang, *Diamond Relat. Mater.*, 2026, **166**, 113791.

13 V. Trivedi, S. Jain, R. Biswas, S. G. Dastider, K. Mondal, S. Bhattacharya, V. Vishal and A. Dutta, *J. Mater. Chem. A*, 2025, **13**, 13378–13390.

14 X. Zhou, C. Meng, W. Yu, Y. Wang, L. Cui, T. Li and J. Wang, *Nanomaterials*, 2025, **15**, 473.

15 Y. Zhao, X. Yu, Z. Wu, Y. Li, W. Wang, L. Feng, Z. Sui and Q. Chen, *Adv. Sustain. Syst.*, 2024, **8**, 2400284.

16 X. Yang, J. Cheng, H. Lv, X. Yang, L. Ding, Y. Xu, K. Zhang, W. Sun and J. Zhou, *Chem. Eng. J.*, 2022, **450**, 137950.

17 S. Li, M. Ceccato, X. Lu, S. Frank, N. Lock, A. Roldan, X.-M. Hu, T. Skrydstrup and K. Daasbjerg, *J. Mater. Chem. A*, 2021, **9**, 1583–1592.

18 J. W. Lim, D. H. Choo, J. H. Cho, J. Kim, W. S. Cho, O. F. Ngome Okello, K. Kim, S. Lee, J. Son, S.-Y. Choi, J. K. Kim, H. W. Jang, S. Y. Kim and J.-L. Lee, *J. Mater. Chem. A*, 2024, **12**, 11090–11100.

19 B. Wulan, X. Cao, D. Tan, X. Shu and J. Zhang, *Adv. Funct. Mater.*, 2022, **32**, 2203842.

20 R. Boppella, M. Austeria P, Y. Kim, E. Kim, I. Song, Y. Eom, D. P. Kumar, M. Balamurugan, E. Sim, D. H. Kim and T. K. Kim, *Adv. Funct. Mater.*, 2022, **32**, 2202351.

21 Y. Li, B. Wei, M. Zhu, J. Chen, Q. Jiang, B. Yang, Y. Hou, L. Lei, Z. Li, R. Zhang and Y. Lu, *Adv. Mater.*, 2021, **33**, 2102212.

22 W. Hua, H. Sun, L. Lin, Q. Mu, B. Yang, Y. Su, H. Wu, F. Lyu, J. Zhong, Z. Deng and Y. Peng, *Chem. Eng. J.*, 2022, **446**, 137296.

23 J. Sun, Z. Liu, H. Zhou, M. Cao, W. Cai, C. Xu, J. Xu and Z. Huang, *Small*, 2024, **20**, 2308522.

24 S. Zhang, P. Yue, Y. Zhou, J. Li, X. Zhu, Q. Fu and Q. Liao, *Small*, 2023, **19**, 2303016.

25 S. Gong, S. Yang, W. Wang, R. Lu, H. Wang, X. Han, G. Wang, J. Xie, D. Rao, C. Wu, J. Liu, S. Shao and X. Lv, *Small*, 2023, **19**, 2207808.

26 S. Lu, M. Mazur, K. Guo, D. C. Stoian, M. Gu, W. M. Tucho and Z. Yu, *Small*, 2023, **20**, 2309251.

27 X. Wang, X. Sang, C. L. Dong, S. Yao, L. Shuai, J. Lu, B. Yang, Z. Li, L. Lei, M. Qiu, L. Dai and Y. Hou, *Angew. Chem., Int. Ed.*, 2021, **60**, 11959–11965.

28 Y. Li, N. M. Adli, W. Shan, M. Wang, M. J. Zachman, S. Hwang, H. Tabassum, S. Karakalos, Z. Feng,

G. Wang, Y. C. Li and G. Wu, *Energy Environ. Sci.*, 2022, **15**, 2128–2140.
